# Stromal Cells Promote Neovascular Invasion Across Tissue Interfaces

**DOI:** 10.3389/fphys.2020.01026

**Published:** 2020-08-14

**Authors:** Hannah A. Strobel, Steven A. LaBelle, Laxminarayanan Krishnan, Jacob Dale, Adam Rauff, A. Marsh Poulson, Nathan Bader, Jason E. Beare, Klevis Aliaj, Jeffrey A. Weiss, James B. Hoying

**Affiliations:** ^1^Advanced Solutions Life Sciences, Manchester, NH, United States; ^2^Department of Biomedical Engineering, University of Utah, Salt Lake City, UT, United States; ^3^Scientific Computing and Imaging Institute, University of Utah, Salt Lake City, UT, United States; ^4^Cardiovascular Innovation Institute, Department of Physiology, University of Louisville, Louisville, KY, United States

**Keywords:** stromal cells, tissue interface, neovessel invasion, vascular biology, VEGF

## Abstract

Vascular connectivity between adjacent vessel beds within and between tissue compartments is essential to any successful neovascularization process. To establish new connections, growing neovessels must locate other vascular elements during angiogenesis, often crossing matrix and other tissue-associated boundaries and interfaces. How growing neovessels traverse any tissue interface, whether part of the native tissue structure or secondary to a regenerative procedure (e.g., an implant), is not known. In this study, we developed an experimental model of angiogenesis wherein growing neovessels must interact with a 3D interstitial collagen matrix interface that separates two distinct tissue compartments. Using this model, we determined that matrix interfaces act as a barrier to neovessel growth, deflecting growing neovessels parallel to the interface. Computational modeling of the neovessel/matrix biomechanical interactions at the interface demonstrated that differences in collagen fibril density near and at the interface are the likely mechanism of deflection, while fibril alignment guides deflected neovessels along the interface. Interestingly, stromal cells facilitated neovessel interface crossing during angiogenesis via a vascular endothelial growth factor (VEGF)-A dependent process. However, ubiquitous addition of VEGF-A in the absence of stromal cells did not promote interface invasion. Therefore, our findings demonstrate that vascularization of a tissue via angiogenesis involves stromal cells providing positional cues to the growing neovasculature and provides insight into how a microvasculature is organized within a tissue.

## Introduction

The process of angiogenesis is fundamental to the formation of new vasculatures during development (Breier, 2000; Nunes et al., 2013), tissue repair (Ravanti and Kahari, 2000), tumorigenesis (Folkman, 1995; Grant et al., 2002), and tissue engraftment (Laschke et al., 2006). One important, largely under-studied aspect of angiogenesis is the process by which growing neovessels navigate through complex tissue structures and stromal compartments in the adult. Here, a neovessel must cross a tissue interface comprised of structured matrix and cells to increase tissue vascularization or to engage with a perfused vascular unit. The latter role of the stroma as a potential barrier to neovessel growth and elongation is particularly relevant when new vascular connections are required to form between adjacent vascular beds, whether present in neighboring tissue compartments or between two distinct tissues. Effective neovascular invasion is perhaps most relevant to the vascularization of implanted tissues. Regardless of the type of implant, the microvasculatures of the implant and the surrounding host tissue must connect across the implantation interface to supply the implant or graft with blood. In the absence of this interface invasion, the implant/graft will become ischemic and fail.

It has been established that stromal matrix deformation, which arises from mechanical loading of a tissue and traction stresses generated by growing neovessels, has a strong influence over neovessel orientation and growth direction (Hoying et al., 2014). This reflects the ability of fibrils comprising the stromal matrix to deform, which is affected by fibril density (e.g., high density reduces compliance and thus deformation), cross-linking (reduces the ability of fibrils to translate and deform relative to each other), and fibril anisotropy (modifies proportion of fibrils engaged in tension). Dense, stiffer matrices promote longer, less branched, neovessels, and retard overall neovessel alignment with more global stromal deformation (Edgar et al., 2014a, b; Underwood et al., 2014). Matrix crosslinks enhance stiffness independent of density yet similarly improve vessel outgrowth and branching (Bordeleau et al., 2017). Fibril alignment similarly affects the microscale matrix stiffness and porosity (Taufalele et al., 2019). Thus, neovessel navigation through tissues likely involves the complex interplay of matrix architecture, mechanical environment, and the spatiotemporal distribution of angiogenic factors. The extent and nature of this interplay has yet to be defined in angiogenesis. Consequently, we explored neovessel guidance dynamics in a simplified model of tissue interfaces involving growing neovessels and a model collagen type I boundary.

To improve tissue vascularization and consequently the success of tissue implants, the mechanisms controlling neovessel invasion across tissue interfaces must be better understood. Toward this, we have created a novel *in vitro* model of a tissue interface, consisting of a high-density collagen layer, formed between two lower density collagen compartments. This system builds on our previous work utilizing intact microvessel (MV) fragments, isolated from adipose tissue, as an accurate *in vitro* angiogenesis model. These MVs, when embedded in collagen, will sprout from the parent fragments, grow, inosculate, and form a neovascular network (Hoying et al., 1996; Nunes et al., 2010). Here, we combined these MVs with our tissue interface system to create an *in vitro* model of neovascular interface invasion.

Surprisingly, growing neovessels do not spontaneously navigate across an interface between two matrix compartments. We determined the role of matrix fibril density and alignment to this neovessel deflection by combining experimental density and alignment measurements at an interface with computational simulations of dynamic matrix:neovessel behavior. Furthermore, our experiments, which mimic the stromal cellular content *in vivo*, identified the importance of tissue stromal cells in enabling angiogenic neovessels to overcome the biophysical cues and invade across the interface. Interestingly, blocking vascular endothelial growth factor (VEGF) produced by the stromal cells abrogated neovessel invasion promoted by the cells. Yet, exogenous addition of VEGF, while stimulating angiogenesis, did not promote invasion in the absence of cells suggesting that spatiotemporal gradients of biochemical cues established by the cells is one means by which the cells promoted invasion. In addition to better understanding how neovasculatures are established in a tissue, these findings provide new insights into the dynamic role of tissue-resident stromal cells in tissue vascularization. These insights may have strong implications in regenerative medicine and angiogenesis-related pathologies.

## Materials and Methods

### Core-in-Field Model of Tissue Interfaces

**FIGURE 1:**
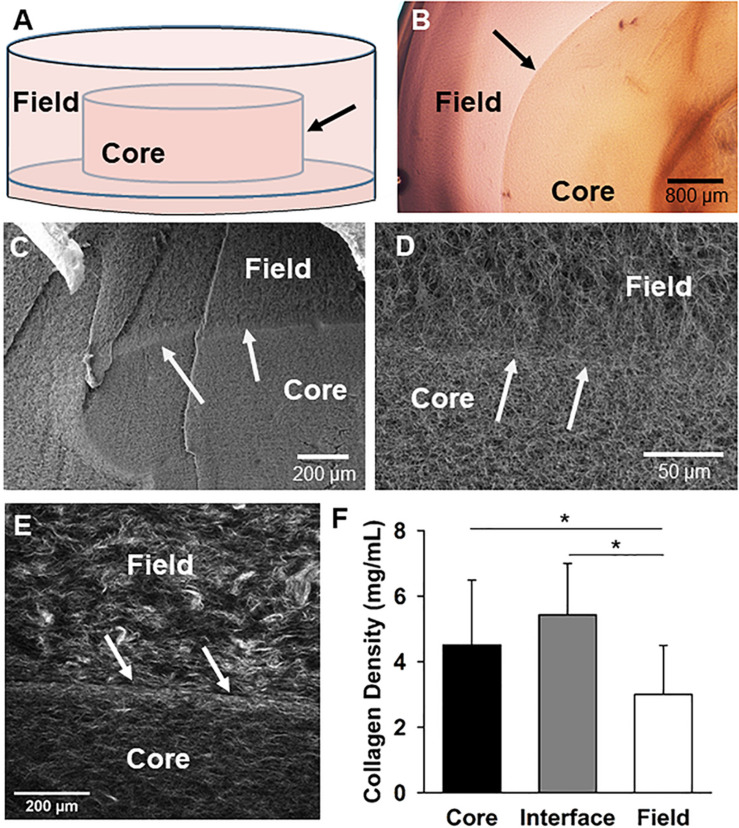
The core-in-field (CIF) tissue boundary model. **(A)** Schematic of the CIF model showing the preformed “core” sitting on a thin bed of gelled collagen surrounded by an additional “field” of gelled collagen. **(B)** Top-view of a phase microscopy image of a microvessel-free and cell-free CIF construct. **(C)** SEM image of a cross-section view of a CIF construct. **(D)** Higher-magnification of the interface and peri-interface region of a CIF construct. **(E)** Second harmonic generation image of the native collagen fibril structure comprising the interface and adjacent regions. **(F)** Fibril densities of the three regions in the CIF construct based on a validated method of measurement from SHG images. Bars are mean ± SD, *N* = 15, one-way ANOVA with Holm-Sidak *post hoc* analysis. *P < 0.05. In all cases, arrows indicate the interface between the core and field.

An *in vitro* analog of tissue-tissue or tissue-implant boundaries was created using a “core-in-field” (CIF) model to examine the extent of vascular sprout outgrowth from the inner vascularized core region to the outer cell free region (Figure 1). Microvessel fragments were isolated from adult Sprague-Dawley rats by limited collagenase digestion and sequential filtration to remove single cells and retain only MVs (Chang et al., 2012). All tissue harvesting was approved by the Institutional Animal Care and Use Committee, and performed following euthanasia. The isolated MVs were suspended in 3 mg/mL collagen solution at a density of 60,000 MVs/mL of collagen solution. This vascularized collagen solution was first pipetted into wells (90 μL/well) of a 96 well plate and gelled to create vascularized cores. The gelled, vascularized collagen cores were then transferred to wells of 48 well plates using a sterile transfer pipet, overlaid with another cell-free collagen solution (250 μL/well), and gelled to envelop the vascularized core completely in cell-free collagen forming a “field” around the vascularized “core.” Dulbecco’s Modified Eagle Medium (DMEM) with 20% fetal bovine serum, 1% penicillin-streptomycin, 1% amphotericin-B was added to each construct once all was gelled (30 min at 37°C). Constructs were evaluated after 9–11 days.

### Preparation and Use of Cells

Rat stromal vascular fraction (SVF) cells were harvested from epididymal fat pads of retired breeder Sprague-Dawley rats, or transgenic Sprague-Dawley rats in which all cells express GFP (SD-Tg(UBC-EGFP)1BalRrrrc; RRRC, Missouri), as previously described (Nunes et al., 2013). Cells were used at 1 × 10^5^ cells/mL collagen unless otherwise stated, which was previously determined as the maximum concentration of cells that did not promote rapid construct contraction (data not shown). Cells were either added with MVs to the construct cores only, or added instead to the field region only of the CIF construct (while MVs remained in the cores).

### Soluble Factors

To test the effect of soluble VEGF on neovessel invasion, a recombinant VEGF-A_165_ (Peprotech) was used at 10 ng/mL final concentration in the media. To trap VEGF in a separate experiment, a combination of recombinant human VEGF R1/Flt-1 Fc Chimera (321-FL/CF, R&D Systems) and recombinant human VEGF R2/KDRFlk-1 Fc Chimera (357-KD/CF, R&D Systems) was used at a final concentration in the media of 1 and 1.5 μg/mL, respectively. The Fc chain of recombinant human IgG1 (110-HG, R&D Systems; 1.5 μg/mL) was used as a control.

### Assessment of Neovessel and Cellular Invasion

Following 9–11 days of culture, the number of neovessels that crossed the interface was counted for each sample. Images of the core were then taken and used to quantify total interface length using ImageJ^1^. Locations where the interface was torn or damaged were excluded from analysis. The number of crossing events for each sample is normalized both to interface length and vessel density. To calculate vessel density, constructs are first stained with Rhodamine labeled Lectin [Griffonia (Bandeiraea) Simplicifolia Lectin I (GSL I, BSL I), Vector Laboratories]. The entire core region was then imaged using confocal microscopy (Olympus FV3000 or Nikon A1R HD), and ImageJ was used to calculate vessel density of the outer region of the core, near the interface. Images were processed to improve contrast, thresholded to identify neovessels, filtered to remove single cells and debris from the foregrounds, and skeletonized. The average neovessel length density (total length of neovessels per area) was then measured using ImageJ. Data are reported for each group as

$$\frac{\#\mathrm{crossing}\mathrm{events}÷\mathrm{interface}\mathrm{circumference}}{\mathrm{neovessel}\mathrm{length}\mathrm{density}\mathrm{in}\mathrm{the}\mathrm{core}}$$

Cellular crossings were calculated by staining constructs with Hoechst dye, and imaging with a confocal microscope. Four images were taken from each sample, one at the top, bottom, left, and right of each sample. The number of nuclei in the field were counted using the cell counter in ImageJ, and this number was normalized to the area of the field region in the image.

All neovessel crossing experiments were repeated 3–5 times, by 2–3 separate investigators, with 3–5 samples per group. Bar graphs are represented as an average of the multiple experiments. A correction factor was employed to account for variation in vessel density between experiments. The corrected vessel density was used to normalize values for crossing events/interface length. In some cases, each experiment was plotted separately on the same graph in addition to overall means.

### Image Acquisition and Collagen Characterization

Second harmonic generation (SHG) imaging was used to quantify collagen fibril density. Second harmonic generation images were acquired using a custom Prairie View Ultima multiphoton microscope (Bruker Corp). Images were acquired with 855 nm excitation and 435–485 nm detection using a high numerical aperture water immersion objective (APO-MP, 25X/1.1W, Nikon). Methods to determine the relationship between SHG signal intensity and collagen density are outlined in Supplementary Figures S1–S3 and Supplementary Data S1.

Scanning electron microscopy (SEM) imaging was performed with FE-SEM/FIB field emission microscope (Tescan Lyra 3 GMU). Samples were cut in half and fixed overnight in 2.5% glutaraldehyde. They were then dehydrated with a graded series of ethanol, then critical point dried and sputter coated with 20–30 nm of gold/palladium prior to imaging.

### Computational Modeling

Microvascular growth and neovessel guidance were simulated to determine the relative contributions of matrix density and fibril alignment to deflection at the interface. Matrix mechanics and the geometry of our experimental setup – two matrix compartments partitioned by an interface – were represented in FEBio, an open-source finite element software^2^ (Maas et al., 2012). FEBio solves for neovessel-induced matrix deformation, which determines local fibril alignment, and stress in the matrix. AngioFE, a plugin that couples neovascular growth and matrix mechanics (Edgar et al., 2012, 2014a,b, 2015a,b), was used in coordination with FEBio to simulate neovessel behavior and crossing at the interface in response to different matrix fibril densities and/or orientations A detailed description of the modeling approach and parametric simulations can be found in Supplementary data S2–S5 and Supplementary Figures S4–S6.

### Statistical Analyses

Statistics were performed using SigmaPlot 11.0 (Systat). One-way ANOVA tests were used on pairwise multiple group comparisons of normal data, with a Tukey or Holm Sidak *post hoc* analysis where appropriate. On data that failed a Shapiro-Wilk normality test, a one-way ANOVA on Ranks with Newman–Keuls or Dunn’s *post hoc* analysis was used. All other pairwise comparisons were analyzed by Student’s *t*-tests. For all statistical comparisons, significance level α = 0.05.

## Results

### The “Core in Field” Model Contains a High-Density Collagen Interface

To investigate the mechanisms of neovessel invasion, we modified our proven isolated-MV angiogenesis model (Hoying et al., 1996; Nunes et al., 2010). In this model, intact MV fragments are embedded within a 3D collagen type I gel. When cultured, neovessels sprout and grow from the individual isolated parent MVs in a way that accurately recapitulates native angiogenesis (Hoying et al., 1996; Nunes et al., 2010; Utzinger et al., 2015). For these studies, we modified this model by establishing a “core” of collagen encased in a surrounding “field” of collagen with either compartment being free of or containing MVs and/or cells. A thin layer of condensed collagen forms as a visible interface between the core and field across which we can assess neovessel crossing/invasion. Construct biophysical features were characterized first without the addition of MVs to the core (Figures 1A,B), by imaging the collagen fibril structure at and adjacent to the interface. Scanning electron microscopy of the collagen structure indicated that the fibril structure from the core, across the interface, and into the field is heterogeneous with differences in both fibril structure and density (Figures 1C,D). Second harmonic generation imaging revealed the interface is comprised of a dense band of collagen relative to the less dense core and field regions, and circumferential fibril alignment at the interface was occasionally observed (Figures 1D–F and Supplementary Figure S7). These differences arose even though the starting collagen concentration used to make both the core and the field was 3 mg/mL.

### Collagen Interfaces Deflect Angiogenic Neovessels, Blocking Interface Invasion

**FIGURE 2:**
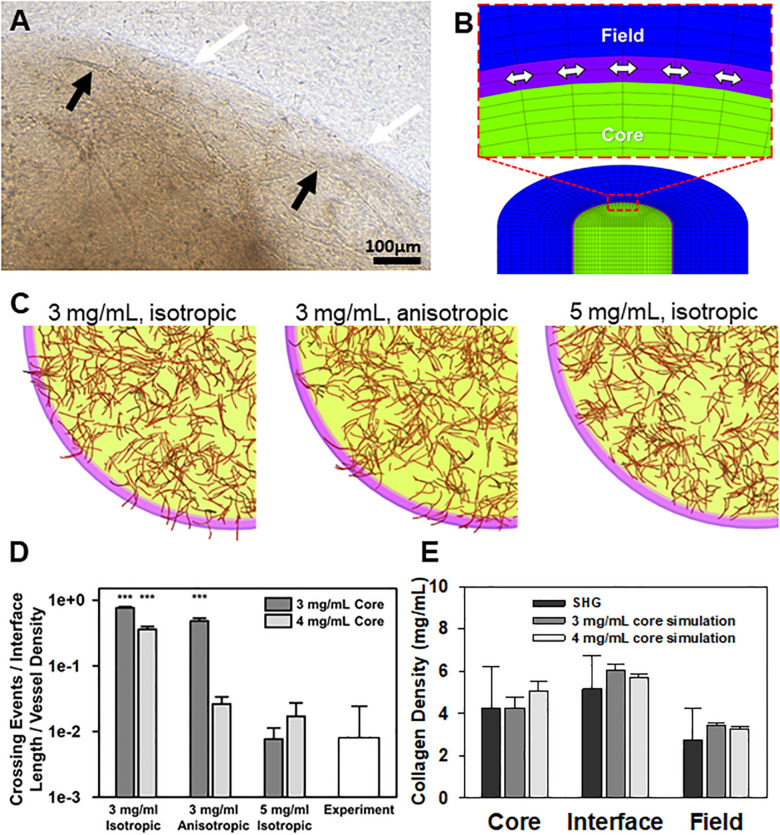
Simulations of neovessel growth in core-in-field models. **(A)** Top-view image of the interface of a CIF construct with microvessels growing along the tissue interface. White arrows point to the interface, black arrows point to microvessel being deflected along the interface. **(B)** Close-up of cut-view of cylindrical CIF geometry used in AngioFE. The core, interface, and field are colored in green, purple, and blue, respectively. Arrows indicate circumferential direction in the interface. **(C)** Simulations of vessel growth and behavior in CIF constructs with different interface densities and fibril organization. Visual results of the simulations for three different conditions of initial interface density (3 or 5 mg/mL) and fibril organization (anisotropic or isotropic) after 10 days of simulated culture. Cores are in light green, interfaces are in pink, fields are clear, and vessels are in red. **(D)** Log plot of predicted neovessel invasion across the interface for the three different simulated conditions, with an initial collagen concentration of either 3 or 4 mg/mL in the core. All groups are compared to the observed experimental value for microvessel-only constructs (white). Bars are mean ± SD, *N* = 4 for experiments, 10 for simulations. One-way ANOVA with Holm-Sidak *post hoc* analysis. ***P < 0.001 compared to experiments. There was an effect of interfacial matrix density on crossing regardless of core density. Further, simulated fibrils were more highly aligned than what was observed experimentally. **(E)** Validation of simulation mechanics determined by predicted final density after microvascular growth. Comparison of day 10 experimental (SHG) collagen densities after microvessel growth and contraction alongside predicted day 10 collagen densities from simulations. Simulations had an initial density in the core of 3 or 4 mg/mL. The initial interface and field densities were 5 and 3 mg/mL, respectively with isotropic fibrils for all simulations. The final densities predicted for each region by simulations are not different from those measured experimentally. One-way ANOVA performed on each region (core, interface, and field). Bars are mean ± SD. *N* = 15 (SHG) or *N* = 10 (simulation). *P* > 0.05.

Surprisingly, when isolated MVs were included in the core at the time of casting and cultured for 7–10 days, growing neovessels were generally unable to spontaneously cross the collagen interface (Figure 2A). As neovessels approached the boundary, they were deflected laterally and continued to grow along the interface, but rarely across it. We have previously shown that growing neovessels are significantly influenced by the mechanics of the stromal environment in a dynamic, reciprocal fashion (Underwood et al., 2014; Utzinger et al., 2015). Specifically, the orientation of collagen fibrils realigns parallel to growing neovessels, the extent of which is affected by fibril density (Krishnan et al., 2008; Underwood et al., 2014). Based on these studies, we next used computational modeling to investigate if the deflection of growing neovessels was due to differences in fibril density and/or fibril alignment at the interface. Fibril alignment was assumed either isotropic or circumferentially aligned along the interface. Matrix density inputs in our models were determined from a multiple linear regression of SHG image intensity and acquisition parameters (Supplementary Figures S1–S3 and Supplementary Data S1). Simulation setup is described in Supplementary Data S2.

In the simulations, neovessels robustly crossed in the absence of a high-density interface regardless of fibril orientation at the interface (Figures 2B–D). In contrast, increasing the interface density to 5 mg/mL reduced crossing to similar levels seen experimentally and, in some cases, prevented all neovessels from crossing the interface. Increasing the initial concentration of collagen in the core from 3 to 4 mg/mL reduced the extent of neovessel crossing (Figure 2D), partially reflecting the effect of higher collagen densities on neovessel growth in the core as well as the increase in collagen density at the interface due to contraction of the matrix by MVs (Edgar et al., 2014b). In all cases, high initial core matrix density or a high-density interface (due to polymerization or MV contraction) was required to prevent crossing. Neovessel mechanics were validated by comparing densities after 10 days of simulation with the experimental densities calculated from SHG image data after 10 days growth *in vitro* (Figure 2E, Supplementary Figure S8, and Supplementary Data S1). The simulations suggest that the condensed nature of the fibril network comprising the interface alone is sufficient to impede and deflect neovessel growth across the interface. Fibril anisotropy may contribute to the reduction in the frequency of crossing but is less potent in preventing invasion than matrix density. Fibril anisotropy does, however, encourage circumferential growth along the interface, a behavior observed experimentally after initial neovessel deflection (Figure 2C center).

### Angiogenic Neovessel Interface Invasion Is Promoted by Stromal Cells

We have previously shown that implantation of MVs embedded in collagen results in rapid inosculation with the surrounding host circulation, progressing to form a new, perfused hierarchical microcirculation (Shepherd et al., 2004, 2007; Gruionu et al., 2010; Nunes et al., 2010). In this situation, the vascular connections with the host circulation have been formed by neovessels that crossed the implant:host tissue boundary. To explain the contradictory lack of neovessel crossing in our *in vitro* system, we considered the possibility that host tissue resident stromal cells at the implant site, which are absent in the *in vitro* CIF interface model, may be critical for mediating neovascular interface invasion in the implants. To test this possibility, we included SVF cells isolated from adipose in our *in vitro* invasion model. We chose adipose because it is a ready source of SVF cells (Williams, 1995; Fraser et al., 2006; Zimmerlin et al., 2010) and SVF can improve lipoaspirate-grafting (Yoshimura et al., 2008), which we reasoned reflects an accelerated integration of graft vessels with the host circulation. Stromal vascular fraction contains the full spectrum of adipose stromal and vascular cells found in adipose tissue.

**FIGURE 3:**
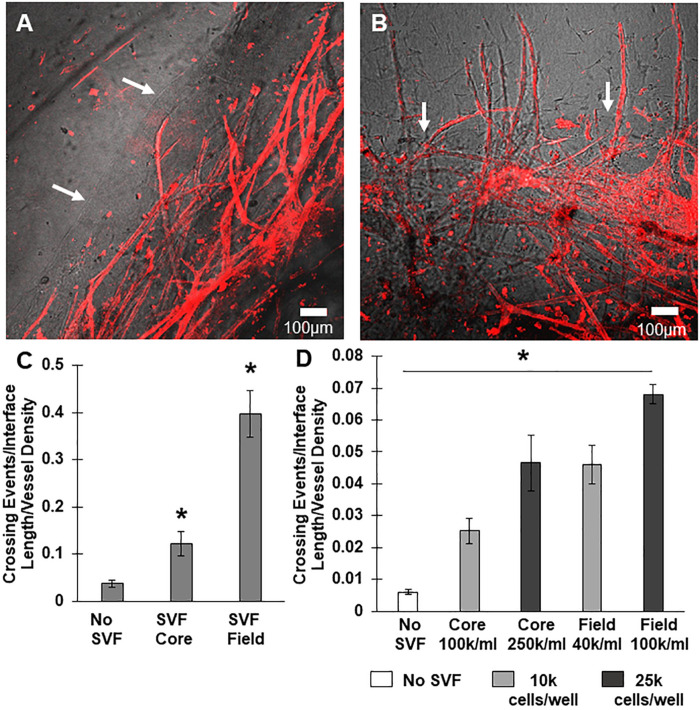
Stromal vascular fraction cells promote neovessel crossing. **(A)** Without SVF cells, microvessels grow up to and then along the interface. **(B)** Inclusion of SVF cells results in neovessels crossing the interface to invade the field region. **(C)** SVF cells resulted in significant increases in neovessel crossing events, particularly when added to the field region. **(D)** Both cell number and spatial positioning affect crossing events. Bars are mean ± SEM, *N* = 4 **(C)** or N = 3 **(D)**. One-way ANOVA with Newman–Keuls **(C)** or Tukey **(D)** *post hoc* analysis. *P < 0.05 compared to all other or specified groups. White arrows point to interface.

We tested two scenarios, one where SVF cells were mixed in the core region with the MVs, and one where SVF cells were located only in the field region adjacent to the MV-containing core. The inclusion of freshly isolated SVF cells in either the core or the field region promoted neovessel interface crossing (Figures 3A–C), although the effect was significantly higher when cells were located in the field region than the core (Figure 3C). As a follow up study, we tested the effect of cell number (Figure 3D). Initially, SVF cells were incorporated at a concentration of 10^5^ cells/mL. However, because the field contains a greater volume than the core, there was a higher total number of cells present when SVF cells were incorporated into the field. To explore the possible effect of cell number, we tested starting SVF cell numbers in the core at both 100k and 250k cells per mL (corresponding to a total cell number of 10k and 25k cells per compartment) and compared that to SVF cells in the field at 40k or 100k per mL (corresponding to 10k or 25k cells per compartment). While increasing the total cell number in the core did increase crossing events, the effect was not as large as having SVF cells in the field. Having 25k total cells in the core was comparable to having 10k total cells in the field (Figure 3D). This suggests that both cell number and cellular spatial positioning have a role in neovessel invasion and guidance during angiogenesis.

### Neovessels and Stromal Cells Do Not Grossly Alter Interface Structure

**FIGURE 4:**
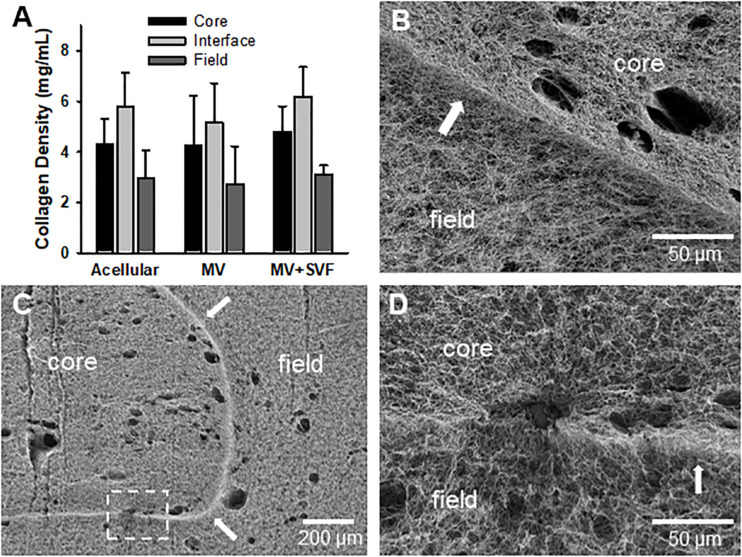
Stromal vascular fraction cells do not disrupt gross fibril structure of the interface. **(A)** Collagen fibril densities at and near the interface as measured by SHG imaging. Acellular CIF constructs are compared to microvessels cultured in CIF constructs (MV) and microvessels and SVF cells cultured for 10 days (MV + SVF). Bars are mean ± SD. *N* = 22, 15, and 10 for acellular, MV, and MV + SVF, respectively. Separate one-way ANOVA for densities in the core, interface, or field, with *P* > 0.05 in all cases. **(B–D)** SEM images of CIF constructs containing either microvessels (MV) or microvessels and SVF cells (MV + SVF). Arrows indicate the interface between the core and field. **(D)** is a higher magnification of the area in panel **(C)** highlighted by the dashed box. Arrows point to interface.

After observing that neovessel crossing is promoted by SVF cells, we began to evaluate possible mechanisms for this effect. We first considered the possibility that the cells remodeled or altered the collagen fibril interface to permit neovessel crossing. However, examination of the interface by SHG microscopy indicated no significant change in gross fibril density due to the presence of growing neovessels and/or SVF cells (Figure 4A and Supplementary Data S1). Nor were there any gross alterations to the interface such as gaps or fragmentation when imaged by SEM, although small holes were visible where cells and/or MVs crossed the interface (Figures 4B–D). Fibril anisotropy for each region (core, interface, and field) did not change with the inclusion of MVs or SVF cells (Supplementart Data S6 and Supplementary Figure S9). All of this suggests that the neovessels and SVF are not profoundly altering interface structure. However, subtle changes to the interface such as clipped cross-links between fibrils or micro-scale gaps between fibril bundles that might promote neovessel crossing cannot be ruled out as these may not be detected by either SHG or SEM imaging.

### SVF Cells Migrate Across the Interface

**FIGURE 5:**
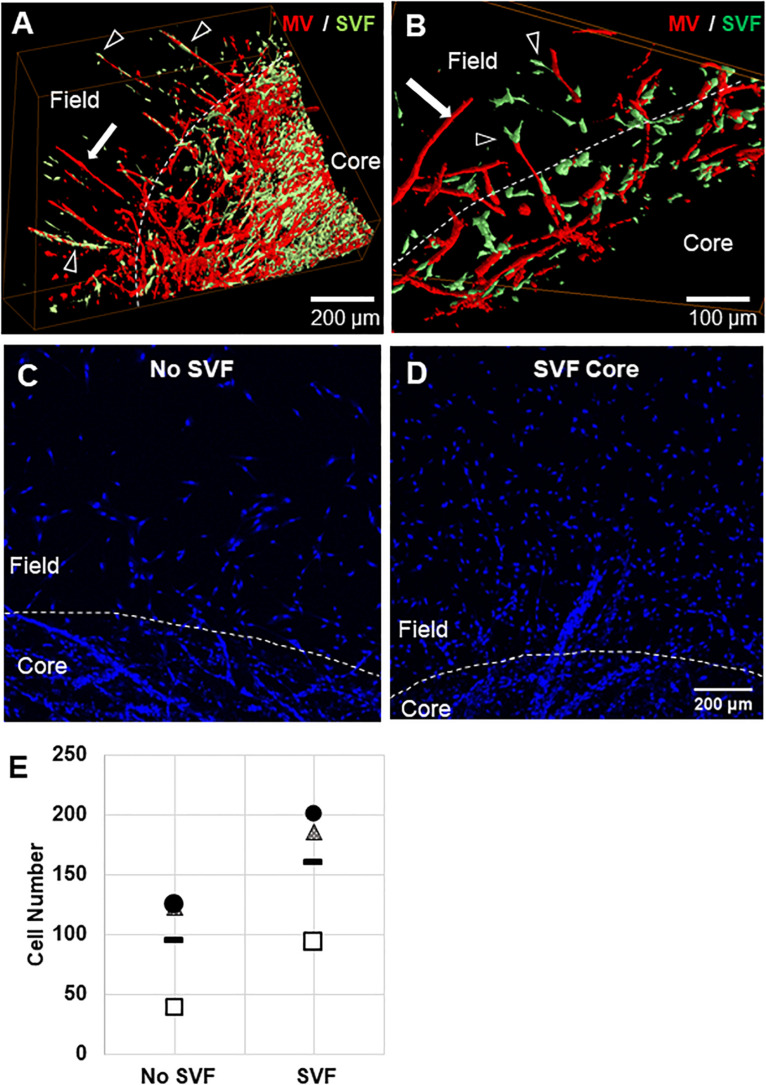
Stromal cell migration from the core to the field. **(A,B)** Confocal image stacks of CIF constructs formed with microvessels (red, rhodamine labeled Lectin stain) and SVF cells (green, GFP+) in the core after 10 days of culture. **(C,D)** Images of Hoechst stained samples without **(C)** or with **(D)** SVF incorporated into the core region. **(E)** Graph of Hoechst stained cells counted in the field region after 10 days of culture, with or without initial SVF incorporation in the core region. The circle, triangle, and box each represent a different experiment, with the line representing the mean of the experiments. Each experiment was statistically evaluated individually using a student’s *t*-test. *P* < 0.05 within the circle experiment and square experiment, but not the triangle experiment. In **(A–D)**, white arrows point to microvessels, black arrows point to SVF. White dashed line indicates the interface.

We also considered the possibility that the SVF cells could promote neovessel invasion by forming new vessel elements across the interface *de novo*, via a vasculogenic-like process. Similar cell preparations can assemble into vessel-like elements when implanted (Koh et al., 2011; Maijub et al., 2014; Ramakrishnan et al., 2016). To test this hypothesis, we incorporated green fluorescent protein (GFP) positive SVF cells into cores with MVs that were not GFP+. Indeed, SVF cells incorporated into growing neovessels, including assembling onto the tip of a growing neovessel (Figures 5A,B). Additionally, there were not neovessels comprised entirely of SVF cells. Importantly, some neovessels that crossed the interface did not have any SVF cells incorporated into their structure. Therefore, while SVF cells do incorporate into growing neovessels, this incorporation does not appear necessary for the pro-invasive neovessel behavior. Interestingly, the findings suggest that in the presence of angiogenic vessels, SVF cells may preferentially add to the growing neovessel instead of *de novo* assembly of neovessels (crossing an interface or otherwise).

To confirm that SVF seeded in the core migrates from the core to the field, we stained constructs with nuclear Hoechst dye and counted cells that had crossed the interface. We previously observed that cells migrate across the interface regardless of presence of SVF, as some single cells remain in the MV isolation, and some leave the MVs after seeding. The number of cells that crossed the interface was much higher when SVF is incorporated within the core than with no SVF (Figures 5C–E). This indicates that the SVF is migrating across the interface, which may be a mechanism for MV crossing events. The cellular composition of SVF is likely different from those single cells migrating off of the individual MVs, which may explain why isolated cells alone do not have this effect while added SVF cells do.

### VEGF-A Mediates Stromal Cell-Promoted Neovessel Invasion

**FIGURE 6:**
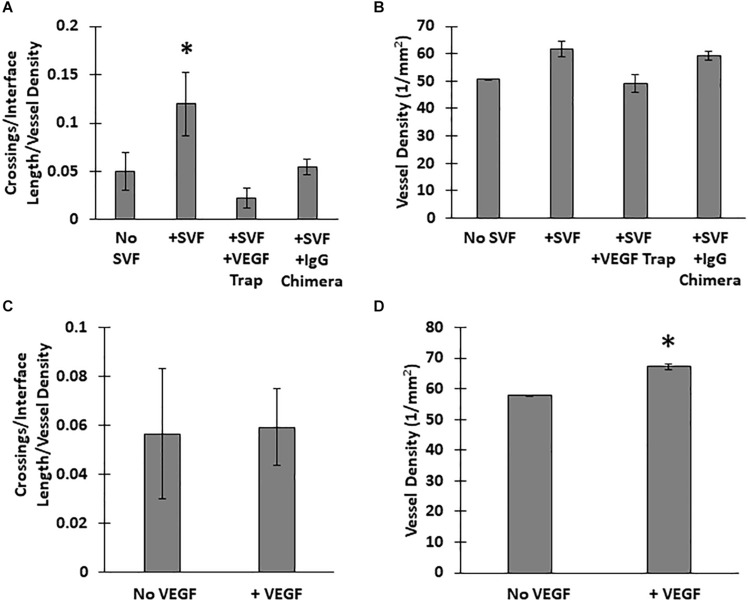
Effect of VEGF-A on neovessel invasion. Effect of a VEGF trap on **(A)** interface crossings and **(B)** vessel density, compared SVF alone and a control IgG chimera protein. **(C)** Normalized neovessel invasion and **(D)** vessel density in CIF constructs containing microvessels alone or microvessels with recombinant VEGF-A_165_ added to the media (+VEGF). One Way ANOVA with Newman–Keuls test **(A,B)** or Student’s *t*-test **(C,D)**. Bars are mean ± SEM, *N* = 5. *P < 0.05 compared to all other groups.

**FIGURE 7:**
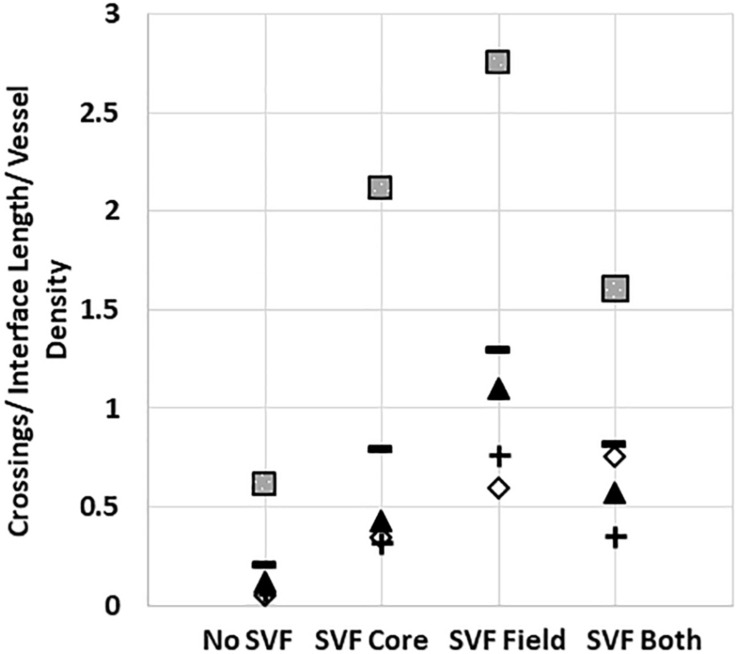
Stromal cells in the core and field together reduce neovessel crossing. Crossing events when SVF is placed in both the core and field, compared to just the core or just the field. Squares, triangles, diamonds and +symbols represent means of 4 different experiments. Dashes represent the means of all experiments. A One-Way ANOVA with Newman–Keuls test was performed on groups within each individual experiment. *P* < 0.05 within the squares experiment for the no SVF compared to both field only and core only; within the triangle experiment for the field compared to all other groups; within the plus experiment for the field compared to no SVF. *P* < 0.05 for the diamond experiment.

Unlike the neovessels, we observed that SVF cells were not impeded by the interface as they readily migrated out of the core into the field. The absence of noticeable interface remodeling by the SVF cells and the irrelevance of cell attachment to the neovessel on pro-invasive activity suggests that the SVF cells provided a guidance cue, enabling the neovessels to overcome the deflective influence of the interface biophysical features. With this in mind, we tested the effect of soluble Flt/Flk receptor chimeras, to sequester VEGF-A, a potent vascular guidance cue (Gerhardt and Betsholtz, 2005). VEGF-A is highly expressed in SVF cells cultured in collagen gels (Supplementary Figure S10). In the presence of the VEGF-A trap, neovessel invasion stimulated by SVF cells was attenuated (Figure 6A), suggesting that SVF mediates neovessel invasion via VEGF-A signaling. While this result was not significant, the VEGF trap brought crossing events close to zero consistently across multiple experiments. The VEGF trap did not have a significant effect on overall vessel growth (Figure 6B). An IgG chimera control for the VEGF trap did show some inhibitory effect on crossing events, but this effect was not nearly as large as the VEGF trap itself. Unexpectedly, when VEGF-A alone (without SVF cells) was added exogenously to MV interface cultures, we did not observe a significant increase in interface crossing, despite the stimulation of higher vessel densities (Figures 6C,D). Thus, despite exogenous VEGF-A promoting angiogenesis in the model, it did not promote more crossing events. These results suggest that it may be a gradient of VEGF-A, rather than its ubiquitous presence, that promotes neovessel interface crossings. To further test this, we compared the presence of SVF in the core or field to SVF in both the core and field. We hypothesized that this would eliminate any gradient of secreted/deposited VEGF between the core and the field, and consequently result in fewer neovessel crossings. We observed that crossings were comparable to the group with SVF in the core only. Having SVF in the field only still had the greatest effect (Figure 7). While sequestration of VEGF influenced SVF cell-mediated neovessel invasion, it is unlikely that VEGF is the sole factor governing angiogenesis in our model as other angiogenic and guidance factors are produced by the MV isolate and SVF cells (Rehman et al., 2004; Altalhi et al., 2017). Furthermore, the VEGF trap employed, which utilizes VEGF-R1 and -R2 ligand binding domains, can also sequester the potent angiogenic placental growth factor (PLGF) (Luttun et al., 2003).

## Discussion

Here, we developed a novel *in vitro* model system for studying neovessel invasion across tissue interfaces. This system utilizes isolated MV fragments, which we have demonstrated can accurately recapitulate native angiogenesis. The model contains two collagen compartments, a “core” and a “field,” with a high collagen density interface layer between them. With this model, we initially observed that angiogenic neovessels are unable to cross the dense collagen interface. This was surprising, given our previous observations that neovessels readily cross such interfaces *in vivo* (Shepherd et al., 2004, 2007; Gruionu et al., 2010; Nunes et al., 2010). Regardless, the finding that neovessels alone do not cross an interface prompted us to explore further the mechanisms that lead to neovessel invasion across tissue interfaces.

We explored the possibility that interface fibril density and orientation influence neovessel crossing behavior similar to that observed with tumor cells invading into matrix compartments (Han et al., 2016). Using SHG imaging, we determined that interface fibrils were compacted relative to those in the core and field. It was not clear, though, if the fibrils were also aligned in any orientation. To separate the potential influence of fibril density from alignment at the interface, we performed computational simulations that model the mechanical interactions between growing neovessels and the deformation of the matrix (AngioFE). When the simulated density (mimicking compaction) of the interface layer between the two compartments was high relative to the bulk core and field compartments, neovessel crossing events were infrequent, matching those observed experimentally. Unlike in the experiments, though, the neovessels did not grow along the interface in this simulation. In contrast, neovessel crossing events in the simulations were more frequent when fibrils at the interface were circumferentially aligned and not compacted. Interestingly, simulated neovessels grew along this aligned interface. It’s important to note that the simulated fibrils were idealized as being entirely aligned at the interface (a likely more extreme case than in the experiments). Overall, our simulations demonstrated that the condensed nature of the fibril network comprising the collagen:collagen interface is sufficient to impede neovessel growth across the interface. Circumferential fibril anisotropy does not appear to be primarily responsible for deflection at the interface but could be involved in directing neovessel growth along the interface. In bulk phase, collagen density and fibril alignment provide contact guidance cues for growing neovessels resulting in differences in neovessel growth rates, branching, and growth direction (Kirkpatrick et al., 2007; Edgar et al., 2014a; Underwood et al., 2014; Utzinger et al., 2015). Our findings highlight different possible roles of fibril alignment during invasion, as well, which may depend on the relative orientation of fibrils at the tissue boundary, cell type, and differences in matrix composition and density on each side of the interface.

Interestingly, the presence of tissue stromal cells (isolated from adipose) promoted neovessel invasion through the interface into the neighboring collagen compartment. This raised the possibility that stromal cells were remodeling the collagen interface such that it no longer served as a deflective barrier to the neovessels. As the density of collagen fibrils is critical in neovessel guidance (Underwood et al., 2014), degradation may facilitate neovessel crossing. When measured, however, collagen density at the interface or areas adjacent to the interface did not change significantly in the presence of MVs, or MVs and SVF cells, over 10 days when compared to acellular constructs. Nor did we observe gross collagen remodeling or degradation by SEM, despite small holes where cells or vessels had crossed. This suggests that neither the MVs nor the stromal cells are grossly degrading collagen fibrils or altering fibril density along the interface. Given the strong dependence of neovessel growth on collagen fibrils, and the absence of gross fibril remodeling, there is likely a different mechanism responsible for neovessel crossing. However, we cannot completely rule out that the SVF made subtle changes to the interface that we were unable to detect. Regardless of whether SVF cell-induced structural changes are present in the interface, some instruction by the stromal cells is required for the growing neovessels to ignore the deflecting contact guidance cues at the interface. For example, cell contraction during migration can form a long-range stiffness gradient in 3D native matrices (Rivron et al., 2012; Han et al., 2018). Such differences in stiffness could further influence fibril-neovessel interactions, affecting neovessel growth and branching during angiogenesis (Edgar et al., 2014b; Underwood et al., 2014). While this is a possible mechanism of instruction by the stromal cells in the bulk phase collagen gel, it seems unlikely that such a phenomenon is directing neovessel growth across the densely packed interface.

Spatially positioned biochemical factors (i.e., gradients) seemed the most likely factor to provide long distance directional cues during angiogenesis. They are known to do this by creating zones of pro-angiogenic signals as well as instructing neovessel orientation via tip-stalk cell dynamics (Gerhardt et al., 2003; Hellstrom et al., 2007; Morris et al., 2015). Furthermore, the matrix-binding ability of nearly all these angiogenic factors results in spatial zones or tracks of angiogenic signals that a neovessel tip cell could detect and track as it moves through tissue compartments. The spatial arrangement of angiogenic factor-driven guidance cues can arise from either matrix architecture (porosity, anisotropy of fibrils and solute diffusion) and/or ligand distribution (heparan sulfate proteoglycans, integrins, etc.). This implies that the sources of these factors within the relevant tissue compartment must be under some local control as the ubiquitous release of factors would not give rise to such gradients, but rather a homogenous distribution. Stromal vascular fraction cells produce angiogenic factors and their heterogeneous spatiotemporal distribution within the tissue may provide the opportunity to create these spatially defined biochemical guidance cues.

Consistent with this idea, sequestration of SVF cell-produced VEGF-A, a known angiogenic factor and guidance cue for neovessels (Gerhardt and Betsholtz, 2005; Ahmed and Bicknell, 2009), largely reduced neovessel invasion. Interestingly, the ubiquitous presence of VEGF-A in the absence of SVF cells was not sufficient to promote neovessel invasion. This suggests that VEGF-A signaling may need to be presented to the growing neovessel as a spatiotemporal gradient (which is not the case when ubiquitously added to the culture) for neovessels to cross the interface; such gradients are necessary in VEGF-A regulated tip-stalk cell behavior (Chappell et al., 2011). The mechanism by which VEGF-A instructs neovessels to overcome collagen fibril durotaxis and contact guidance and cues at the interface is unclear. Whether VEGF-A regulates proliferation or migration, both of which are necessary for angiogenesis (Rauff et al., 2019), during interface crossing is unclear. Given that exogenous VEGF-A promoted overall angiogenesis in our model, and thus both proliferation and migration, but not neovessel guidance, it is likely another VEGF-dependent activity is relevant. One possibility is that differential signaling by VEGF-A receptors may be playing a role. For example, deletion of the neuropilin-1 (Nrp-1) gene in mice results in the inability of neovessels to cross laterally into neighboring tissue compartments in the developing mouse hindbrain (Gerhardt et al., 2004), a tissue dynamic similar to that modeled in our experiments. NrP-1 is a receptor expressed on neovessel tip cells that binds the VEGF-A_165_ isoform, contributing to neovessel guidance in coordination with VEGF Receptor 2 (VEGFR2; Gerhardt et al., 2003; Zachary, 2011). Alternatively, changes in matrix architecture near the interface may induce differential mechanoregulation of VEGF signaling members. Sprout polarization and reorientation in response to VEGF signaling slows with increasing collagen density unless a higher magnitude VEGF gradient is introduced (Shamloo et al., 2012). Such a phenomenon could partially explain the different magnitude in crossing observed when SVF cells are included in the field rather than the core. Fibril alignment secondary to matrix deformation has been shown to increase VEGFR2 expression, potentially enhancing the angiogenic response of neovessels (Rivron et al., 2012). Whether similar dynamics are contributing to neovessel interface deflection and invasion in our system remains to be determined.

**FIGURE 8:**
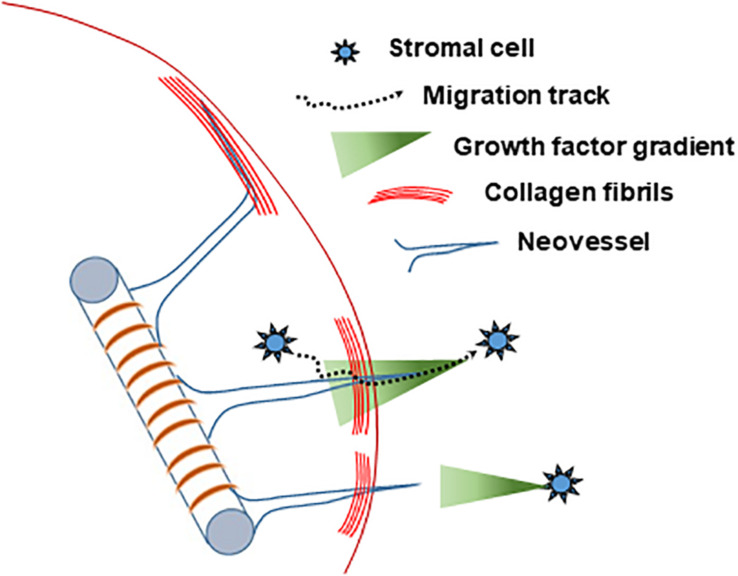
Schematized hypothesis of how stromal cells guide growing neovessels across a tissue interface regardless of location relative to the growing neovessels.

Based on our collective findings (summarized in Table 1) and the above discussed considerations, we propose the following working model of neovessel invasion across tissue interfaces (Figure 8). Stromal cells, which freely move between tissue compartments, establish spatial gradients of VEGF, which guide neovessels across the interface. A key element of this hypothesis is the spatiotemporal position of the stromal cell relative to the neovessels and the interface, and, consequently, the spatiotemporal orientation of a VEGF-A guidance cue. When stromal cells are located opposite the growing vessels (i.e., in the field), soluble VEGF-A will naturally form a gradient as it diffuses through the collagen toward the core. When stromal cells are in the core only, the cells may be secreting or depositing a gradient of VEGF-A as they migrate through the interface and into the field. This cellular migration was confirmed via Hoechst staining, which showed a larger number of cells migrating into the field when SVF was seeded in the core, compared to controls without SVF. A larger increase in neovessel crossing events was observed when stromal cells were in the field region, rather than mixed together with MVs in the core, suggesting a large diffusion guidance cue from the field SVF exists. This large field-derived diffusion gradient is decreased when cells were seeded on both sides of the interface. This leaves only local gradients established by cells migrating from the core into the field. As only a portion of the cells in the core will cross the interface, the total crossing event is fewer than when SVF is in the field only. Therefore, there are likely two types of guidance cues derived by stromal cells: large angiotactic gradients that guide over longer distances and local cell-derived guidance tracks. Given that VEGF-A is present as different isoforms with differing abilities to bind matrix, it is likely that gradients consist of both bound and freely diffusible VEGF-A. Each aspect of these gradients may potentially contribute differently to the guidance cues sensed by the neovessels, thus gradients produced in the core and field may not have equal effects.

**TABLE 1:** Summary of neovessel invasion findings.

Experimental condition	Key observations	Conclusion/Implication
Neovessels alone	Neovessels do not cross interface	
Neovessels + SVF in core	Neovessels cross interface	Stromal cells promote neovessel invasion
Neovessels + SVF in field	Neovessels cross interface	

SVF in collagen gels	Express VEGF-A	VEGF-A presence is necessary but not sufficient for SVF-enabled neovessel invasion
Neovessels + SVF + anti-VEGF	Neovessels do not cross interface	
Neovessels + recombinant VEGF	Neovessels do not cross interface

SHG of fibril architecture	Fibrils at interface are dense	Deflection of neovessels at the interface is due to collagen fibril density at the interface.
Simulation: increased interface density	Neovessels do not cross interface	
Simulation: fibril alignment at interface	Neovessels cross interface without high initial density

Tissue vascularization is important for ischemic tissue repair, wound healing, regenerative medicine, and in many pathologies. In all cases, tissue boundaries exist that could impede integration of the new vasculature into the existing circulation. This is particularly relevant in applications involving grafts and tissue implants where discrete implant and recipient-site boundaries are present. Consequently, solutions facilitating the guidance of neovessels across tissue interfaces would significantly advance vascularization-dependent therapies and tissue vascularization strategies. Our evidence suggests that the stromal cells, normally resident within tissues, play a key role in promoting tissue vascularization and could be leveraged to facilitate vascularization, or, alternatively, targeted to stop vascularization in pathology. In fact, many of the cells used in adult cell therapies, due to pro-vascular capabilities intrinsic to the cells (Moon et al., 2006; Traktuev et al., 2008; Morris et al., 2015), promote tissue vascularization. In this context, the core in our model represents an idealized tissue implant in which a preformed, angiogenic vasculature is implanted into a relatively low density, quiescent vascular environment. This is because angiogenic vascular beds tend to produce a high number of neovessels, which will over time be pruned and remodeled as the vascular bed matures. In the case of our CIF, we have simplified the model to a more extreme scenario where the implant site (i.e., the field) is avascular. Our results suggest that incorporation of stromal cells in the tissue implant (i.e., the core) and/or delivery of stromal cells to the implant site (i.e., the field), whichever is more feasible, would facilitate vascularization and engraftment of the implant.

## Data Availability Statement

The raw data supporting the conclusions of this article, as well as the input files used for the simulations, will be made available by the authors, without undue reservation. The open-source FEBio software used for the simulations is available at www.febio.org. The AngioFE plugin for FEBio used for the simulations is available at www.febio.org/plugins/.

## Ethics Statement

The animal study was reviewed and approved by Dartmouth College IACUC.

## Author Contributions

LK and JH designed the core-in field model. HS and JH designed the experiments. HS, JD, LK, NB, and JB performed the experiments and data analysis. NB developed the image analysis algorithms used to assess microvessel density. AR, HS, KA, and SL performed the structural imaging and analysis. AP, KA, SL, and JW designed the computational framework. SL designed and performed the computational simulations. LK, SL, HS, AR, JW, and JH wrote and edited the manuscript. All authors contributed to the article and approved the submitted version.

## Conflict of Interest

JH is a partner at Advanced Solutions Life Sciences, which commercializes the microvessel technology. The commercial activity of ASLS is outside the scope of the submitted work.

The remaining authors declare that the research was conducted in the absence of any commercial or financial relationships that could be construed as a potential conflict of interest.

## Footnotes

1 https://imagej.nih.gov/ij/

2 www.febio.org

## Abbreviations

ANOVA: Analysis of Variance
CIF: core-in-field
DMEM: Dulbecco’s Modified Eagle Medium
GFP: green fluorescent protein
MV: microvessel
SD: Standard Deviation
SEM: scanning electron microscopy
SEM: standard error of the mean
SHG: second harmonic generation
SVF: stromal vascular fraction
VEGF: vascular endothelial growth factor.
