# Supplementary material for: Stromal Cells Promote Neovascular Invasion Across Tissue Interfaces
Source: Front Physiol. 2020 Aug 14;11:1026. doi: 10.3389/fphys.2020.01026 (PMC7461918; doi:10.3389/fphys.2020.01026)
Supplement: Supplementary file 1 [file Data_Sheet_1.pdf]

Supplemental Material

1 Supplemental Methods

1.1 Supplement S1: Quantification of collagen density from signal intensity of Second Harmonic Generation (SHG) images

Motivation:
To approximate the quantitative relationship between signal intensity and collagen density to determine measures of effective collagen density in each region of the core in field gels.

Methods:
Acellular gels were prepared using the protocol mentioned in the methods. These control gels contained one compartment. Gel concentrations were 1, 3, 5, 6 mg/ml. Phosphate buffered saline was imaged as a 0 mg/mL analog. SHG Images were acquired using a custom Prairie View Ultima multiphoton microscope (Bruker Corp., Billerica, MA, USA). Images were acquired with 855 nm excitation and detection in 435 – 485 nm. A high numerical aperture water immersion objective was used (APO-MP,25X/1.1W, Nikon). Image intensity was converted to a double precision number and mapped to range [0 1]. The photomultiplier and laser attenuation were varied to obtain sufficient image contrast (**Supplemental Figure S1**). A 4 (PMT values of 550, 675, 750, 800) x 3 (attenuation values of 17.995, 18.995, 19.995) two-way ANOVA was performed to check for interactions between input parameters. Three gels of each concentration were made and 6 images were taken from each gel at a given acquisition setting. The appropriate number of images to stabilize the moving standard deviation was determined to be 6 (**Supplemental Figure S2)**. A multiple linear regression was prepared using SigmaPlot11.0. The intercept term of the regression was adjusted so the average of all field measurements from avascular core in field gels was 3.0 mg/ml since we expect this region to change the least with respect to the initial density.

Results:
The two-way ANOVA identified significant main effects (PMT: $P < 0.001$, $F = 10129.444$; Laser power attenuation: $P < 0.001$, $F = 160.083$) and a significant interaction between PMT and laser attenuation percentage ($P = 0.030$, $F = 12.837$). N = 6 for each assessed combination of PMT and laser attenuation percentage. The resulting multivariate fit was

$$\boldsymbol{CD} = 12.946\boldsymbol{M} - 0.05556\boldsymbol{L} - 0.0139\boldsymbol{P} + 12.278 \quad \textbf{(S6)}$$

$R^2 = 0.906$
CD – Collagen Density
M – Mean Image Intensity
L – Laser Power Attenuation Percentage
P - Photomultiplier

Conclusion:
All independent variables significantly contributed to the dependent variable ($P < 0.001$). A negative relationship between collagen density, PMT, and laser attenuation percentage was

expected since increasing these parameters yields higher intensity while collagen density remains unchanged. The regression's variance increased with distance from 3 mg/mL concentrations (**Figure S3**).

1.2 Supplement S2: Simulation of microvascular growth and guidance

AngioFE growth simulation

To explore whether variations in collagen fibril orientation could deflect neovessels at the interface, we performed simulations in AngioFE 2.0 as a plugin for FEBio 2.10. AngioFE2 is available as an executable at https://febio.org/plugins/ or as source code at https://github.com/febiosoftware/AngioFE2. FEBio 2.10 can be downloaded at www.febio.org/downloads or https://github.com/febiosoftware/febio2.

Model Geometry

The geometry was based off the core in field constructs. The core (radius 1.96 mm), interface (radius 1.96 – 2.04 mm), and field (radius 2.04 – 5.00 mm) were discretized into separate materials and had a height of 4.42 mm.

AngioFE growth model inputs

Growth Length

The network length over time in our *in vitro* models follows a sigmoidal curve. The growth length over time for a single fragment was thus determined from the time derivative of the fit to the sigmoidal curve

$$g(t) = g_0 + \frac{a_g}{1 + e^{-\frac{t - t_{1/2}}{b_g}}} \quad \textbf{(S1)}$$

The derivative of this is thus

$$\frac{dg(t)}{dt} = \frac{a_g e^{-\frac{t - t_{1/2}}{b_g}}}{b_g \left(1 + e^{-\frac{t - t_{1/2}}{b_g}}\right)^2} \quad \textbf{(S2)}$$

The magnitude of the curve for an individual sprout is determined as the total vascular growth at day 10 divided by the initial number of tip cells. For our models, the initial number of fragments determined from the seeding density and core volume is 3,000 fragments; thus, there are 6,000 tips (2 tips per fragment). a_g was determined from prior published growth curves divided by 2 due to changes in code inputs i.e. 568.6/2=284.3.

Table S1: Segment growth parameters

Parameter	Value	Units
a_g	284.3	µm/tip
b_g	1.3	days
$t_{1/2}$	4.8	days
g_0	42	µm

Loadcurve 1 in the input files is specified by Eqn S2 with parameters from Table S1. This load curve prescribes the length of additional segments over time before scaling parameters such as those from local density are applied.

Branching
Branching was simulated by prescribing the length to branch and the time to emerge. Branch points, i.e. points along a vessel where a branch could form, were determined with a narrow normal distribution with mean and standard deviation 315 ± 1 µm. The actual time at which a branch formed from a branch point was then determined from a uniform distribution for the entire simulation duration.

Neovessel Orientation
We have previously predicted neovessel growth and alignment (Edgar et al, 2014 and Edgar et al 2015). At each new time step the direction of new neovessel growth $\boldsymbol{\psi}_{t+1}$ is determined as a weighted sum of the previous growth direction $\boldsymbol{\psi}_t$ (persistence component) and the local collagen fibril orientation $\boldsymbol{\theta}_t$ vectors:

$$\boldsymbol{\psi}_{t+1} = \alpha\boldsymbol{\theta}_t + (1-\alpha)\boldsymbol{\psi}_t \quad \textbf{(S3)}$$

Here, α is a weighting parameter with a value between 0 and 1 where 0 indicates complete dependence of growth on the previous direction and 1 indicates complete dependence of growth on the local fibril orientation. Previously reported values of $\alpha = 0.91$, the dependence of neovessel orientation from our publications, were time step dependent. The updated version of the plugin has removed this dependence and the new parameter that provided the same deformation for previous models was determined at $\alpha = 0.36$.

Mechanics simulations

The mechanics generally followed those outlined previously (Maas et al, 2012). FEBio 2.10 and AngioFE2 were used for all simulations (www.febio.org). Deviations from previous publications are as follows:

Sprout Body Stress
The sprout stress magnitude is assumed to increase in a similar manner as the vascular length based on observations that core in field gel contraction and cell growth remain low until day 6 before contractions and growth increase. The stress was prescribed as

$$f_b(t) = \frac{a_f}{1+e^{-\left(\frac{t-t_{1/2}}{b_f}\right)}} \quad \textbf{(S4)}$$

Equation S4 was designed to follow experimental observations of gel contraction; a narrow transition region accounts for strong contractions seen between days 6 and 8 *in vitro*. Previously the sprout magnitude per single sprout was determined at 3.72 x 10^{-6} Pa (Maas et al 2012). The value chosen for a_f in the core in field model is slightly lower but within the range previously studied. Load curve 3 in the input files corresponds to Eqn S4. Note that units in the input file are in base units of μm for length and days for time, thus stress in the input files are in units of kg·μm/day^2.

The parameters used are summarized in Table S2:

Table S2: Sprout stress parameters

Parameter	Description	Value	Units
a_f	Sprout stress magnitude	3.72	μPa
$t_{1/2}$	Center of sigmoidal curve	7	days
b_f	Control width of force sigmoidal curve	0.5435	days
b	Sprout stress range	250	μm
N	Sprout stress width	2	[ ]

Time-stepping scheme

AngioFE and FEBio used different time steps. At each 0.1 day increment, AngioFE would simulate neovessel growth and update the forces at a sprout tip. To reduce computational demand, FEBio was updated at 0.5 day increments. FEBio applied sprout stresses from AngioFE then determined the resulting deformation at each increment.

Material Parameters

The extracellular matrix and vessel passive mechanics were modeled as before. The same values previously used were chosen and are presented in Table S3 (Maas et al, 2012).

Table S3: Material parameters

Parameter	Description	Value	Units
E_{M}	Ground matrix stiffness	35.862	Pa
E_{fib}	Collagen fibril stiffness	358.62	Pa
E_{vess}	Vessel stiffness	3,586.2	Pa
τ	Viscoelastic time constant	1.08	s

Simulation Deformation Evaluation:

SHG images were generally taken 100 - 300 μm below the surface of the gel. The top and bottom of the gel were imaged. Images were restricted to within 400 μm of the interface. Thus, the densities calculated from the simulations for each region was calculated from elements in similar geographic positions (**Figure S8**).

Simulations of Spatial Variation in Density

SHG measurements of collagen gel density demonstrated that the interface between the core and field was composed of much higher density collagen. Further, the density of collagen in the core changed during the period of culture. We performed simulations to determine if these spatiotemporal variations in collagen density could be responsible for neovessel deflection at the interface. In AngioFE, the rate of neovessel growth and sprout body stress are scaled by the local collagen density Eqn S5 (Edgar et al, 2014).

$$v = v_0 + a_0 \exp(-a_1 c) \quad \textbf{(S5)}$$

Here, v is a scaling factor, c is the collagen density in mg/mL, and the other terms were fit from prior experiments growing neovessels in collagen gels of 2, 3, or 4.5 mg/mL. A study was performed to determine the optimal meshing near the interface to accurately reproduce the local density **(Supplement S4; Supplemental Figure S5)** and changes in local fibril alignment **(Supplement S5; Supplemental Figure S6)**. Further, growth of a tip cell was ended if the calculated new segment length was below 0.001 μm. Thus, simulated vessels either change direction in response to density gradients, (persistence in the direction of high densities will decrease) or stop growing. We simulated 3 different core-in-field conditions: 1) random orientation of fibrils in the core, field, and interface but increased density (5 mg/mL) at the interface, 2) circumferentially aligned fibrils along the interface, and 3) random fibril orientation in the absence of a density gradient. Each condition was simulated with the core collagen density prescribed at either 3 or 4 mg/mL based on the range of measured collagen density. Ten simulations were run for each condition and density, randomly varying the initial positions of the parent neovessels in the core.

Simulations of Anisotropy in the Tissue Interface

Both two and three dimensional alignment at the interface were studied. For the case of 2D alignment, fibrils were oriented in the circumferential direction and contained no vertical component. For the case of 3D alignment, fibrils were oriented in the circumferential direction then given a vertical component based on sampling from various orientation distribution functions (**Supplement S3; Supplemental Figure S4**). We also investigated the effect of the number of interfacial elements and the interface thickness (**Supplemental S5; Supplemental Figure S6**). The density was set at either 3 or 4 mg/mL for all regions to account for the range of densities observed in the core and field via SHG. Ten simulations were run for each density with parent microvessels seeding locations randomized in the core each time. To determine the crossing events/interface length/vessel density, the total number of events per model was first divided by the perimeter of the entire interface. The vessel density was first determined as the total number of vessels in the volume of the core which was then scaled by the image depth used to acquire epifluorescent images so that the vessel densities would be comparable. Finally, the crossing events/interface length was divided by this comparative vessel density.

1.3 Supplement S3: Anisotropy study – Three-dimensional orientation of tangential fibrils at the interface

Motivation:
Fibrils in the case of complete alignment were assumed to all lie within the horizontal plane. It is likely that fibrils or fibril families at the interface could be tangential to the interface but contain a vertical component as well. Distributions of fibrils tangent to the interface but rotated some degree with respect to the radial direction axis were examined to determine the sensitivity of vessels to the axial component of the fibril orientation at the interface (**Figure S4A**).

Methods:
Initially fibrils were assumed to remain tangent to the interface with components only in the horizontal directions. To study other distributions of fibril alignment at the interface, the tangential direction was rotated about the radial position vector at an angle determined by various von Mises distributions (**Figure S4A**). Four distributions of vertical rotation were examined in addition to tangential (no vertical component) (**Figure S4B**) – a uniform distribution (rotation at all angles equally likely), horizontal bias, vertically biased, or a 2 fiber family distribution angled at $\pm 45^o$ (cross distribution). The density was set to 3.0 mg/mL for each region. Ten simulations (each with different random samplings from the chosen von Mises distributions) were run and averaged for each distribution. The dependence of growth direction on fibril direction was set to values used previously ($\alpha = 0.36$). Differences were analyzed with a One-way ANOVA and Holm-Sidak all multiple comparison procedures test in SigmaPlot 11.0.

Results:
There was a difference in total crossings between groups ($p = 0.008$, $F = 3.923$, dof = 49 overall). This difference is limited to tangential and cross fiber distributions (APMCP $p < 0.001$, $t = 3.617$; **Figure S4E**). Thus, the three-dimensional orientation of fibrils in the interface is predicted to play a role in the success of neovessel crossings at the interface for certain fibril orientation distributions.

Conclusion:
Simulations of two of the fibril distributions predicted increased crossing (~11 %) compared to complete alignment in the horizontal plane. Simulations of vessel crossings can be sensitive to the three-dimensional orientation of fibrils.

1.4 Supplement S4: Mesh study – Collagen density distribution

Motivation:
Vessel growth and force are dependent on the local collagen density. Thus, it is important to ensure that the chosen mesh is able to accurately represent the near discontinuous rise in collagen density near the interface. A mesh study was performed to determine the effect of meshing near the interface within the core on the observable collagen density as a function of radial distance. Mesh resolution and radial biasing were varied for a total of 6 meshes denoted as low, medium, or high meshes that were biased or nonbiased towards the interface based on the number of elements in each region (**Figure S5A**).

Meshing Schemes:
A butterfly mesh was used for the cylindrical core. A central 20 x 20 rectilinear core was used across all models. This resolution extends to the circular region of the core mesh and the tubular interface and field, providing an angular resolution of 4.5 °/element. An axial (z-direction) resolution of 22 elements (~200 μm/element) was used for all meshes. The interface was meshed as a 2-element, 80 μm thick tube centered at 2,000 μm for all cases based on the interface anisotropy mesh. Three radial meshing densities were considered with unbiased and biased meshing schemes. The low-resolution meshes consisted of 5 radial elements in the circular region of the core mesh and 10 radial elements in the field. Medium-density meshes increased the number of core radial elements to 10 while maintaining 10 elements in the field. High-density meshes increased the radial element density to 15 for both the core and field. The initial density of the core and field were 3 mg/mL and the initial density of the interface was 5 mg/mL.

Mesh Assessment:
Network length quantifications for each mesh were approximately equal within the core but did increase with mesh density and biasing (**Figure S5B**). The high biased mesh best represented the steep change in collagen density between the core and the interface (**Figure S5C**) whereas the other schemes extend the range of the density gradient resulting in slightly lower growth in the core and interface.

The collagen density at the final step was averaged about the initial radial position to compare material points initially sharing axisymmetric properties (**Figure S5C**). Lower refined and unbiased meshes exaggerated the effective radius of the interface by creating a wider observable region of high-density collagen.

Conclusion:
The high, biased mesh was selected due to its enhanced resolution near the interface and observable effect on vessel growth in the interface without affecting morphometrics within the core.

1.5 Supplement S5: Mesh study – Two-dimensional tangential interface anisotropy

Motivation:
As simulated neovessels grow, it is possible for them to grow through more than one element if the element thickness in the direction of growth is small enough. To account for the possibility that neovessels could grow through the thin elements at the interface (and thus “miss” local data that may affect growth e.g. density, fibril alignment), a mesh study was performed examining the total thickness of the interface and number of radial interfacial elements needed to deflect neovessel growth.

Meshing Schemes:
The core and field regions were meshed with the high biased mesh from the density mesh study. The interface thickness and number of elements were varied at 30, 80, and 130 μm and 2, 3, or 4 radial elements. Fibril orientation for all interfacial elements was prescribed tangent to the circumferential direction within the horizontal plane to represent an idealized case of complete anisotropy tangent to the interface (**Figure S6**). The collagen density was set to 3 mg/mL for all regions. The growth model in AngioFE was adjusted so that the current growth direction depended entirely on the local fibril direction ($\alpha = 1.0$); thus, an appropriate selection of interface thickness and meshing density should predict few vessel crossings into the field. The total growth in the field was quantified to identify a sufficient thickness and meshing density in the interface.

Mesh Assessment:
Crossings were only predicted for a 30 μm interface; no crossings were predicted otherwise.

Conclusion:
The 80 μm, 2 element mesh with idealized fibril alignment and growth along fibril directions was capable of completely deflecting vessel crossings and thus was chosen for successive studies. This thickness was also closest to those measured from SHG images.

1.6 Supplement S6: Collagen hydrogel anisotropy from Second Harmonic Generation (SHG) images.

Motivation:
We sought to determine if fibril anisotropy was present at the interface after polymerization and after 10 days of growth. We were also interested in determining if there were any changes in anisotropy over time as a result of growth.

Methods:
Core in field gels were made either without cells, with MVF, or with MVF + SVF in the core. Avascular gels were fixed after polymerization. Gels containing MVF were fixed after day 10. MVF + SVF gels were fixed at either day 0 or day 10 to determine if the inclusion of SVF altered the starting orientation of fibrils at the interface or if the inclusion of SVF resulted in changes in orientation after culture. The field of view and microscope settings were chosen so the core, interface, and field are visible (**Figure S7**). Cropped images pertaining to each region were then processed using a custom MATLAB script. Anisotropy was determined as Anisotropy $= 1 - (\lambda_1 / \lambda_2)$ where λ_1 and λ_2 are the eigenvalues for the covariant matrix obtained from the power spectrum of the fast Fourier transform image. Here, a measure of 1 indicates full alignment and 0 indicates no alignment. At least 15 images for each region were acquired from avascular, MV, or MV + SVF gels after culture. A two-way ANOVA was performed to assess variations in anisotropy between each region (core, interface, field) and each treatment (avascular, MV, or MV + SVF). A Holm-Sidak all pairwise multiple comparison procedures test was used to identify groups that differed significantly.

Results:
Orientation varied between isotropic and aligned (**Figure S7**). Orientation was analyzed with a 3 (Core, Interface, Field) x 3 (Avascular, MV, MV + SVF) 2 way ANOVA (N = 23, 31, and 15 per region for avascular, MV, or MV + SVF respectively) (**Figure S9**). The main effect of region was significant ($P < 0.001$, $F = 20.070$), the main effect of cell composition was insignificant ($P = 0.489$, $F = 0.718$), and there was no significant interaction between region and cell composition ($P = 0.607$, $F = 0.680$). A Holm-Sidak all pairwise multiple comparison procedures indicated that anisotropy decreased in the field relative to the core and field. Because there was no significant difference between treatments or interaction we do not detect differences in fibril anisotropy due to the inclusion of MVF or MVF + SVF. The least square mean for all regions were below 0.48, indicating moderate alignment.

Conclusion:
A moderate degree of anisotropy was found in each region near the interface. Anisotropy in the core and interface differed significantly from the field. Anisotropy did not change with the inclusion of different cell fractions.

2 Supplemental Figures

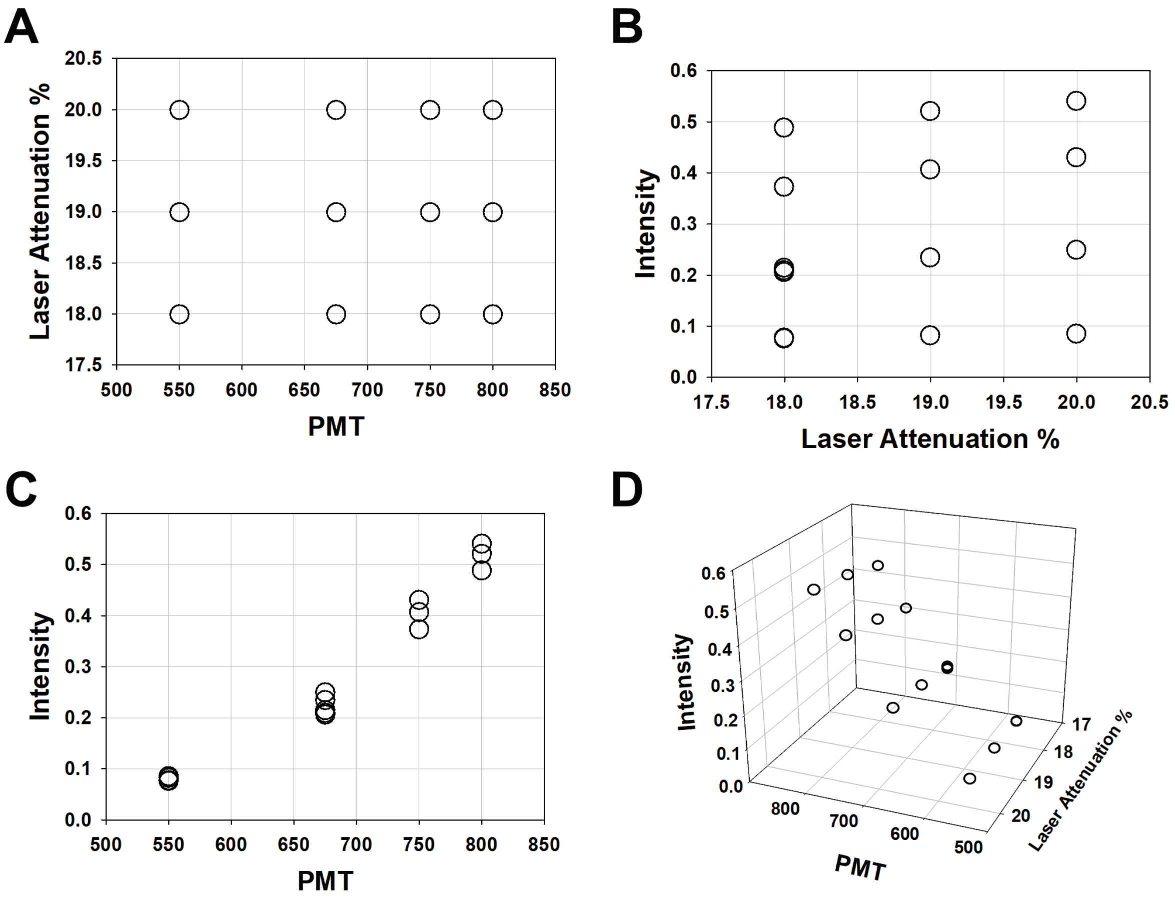

<u>Supplemental Figure S1.</u> Effect of PMT and laser attenuation settings on image intensity. Photomultiplier (PMT) and laser attenuation percentage were adjusted until the image exhibited appropriate contrast and clarity. Images were acquired with the settings plotted. **A**) The PMT ranged from 550 – 800, and laser attenuation percentage varied from 18 - 20%. **B-D)** The average image intensity was computed for each image, and plotted against PMT and laser attenuation percentage.

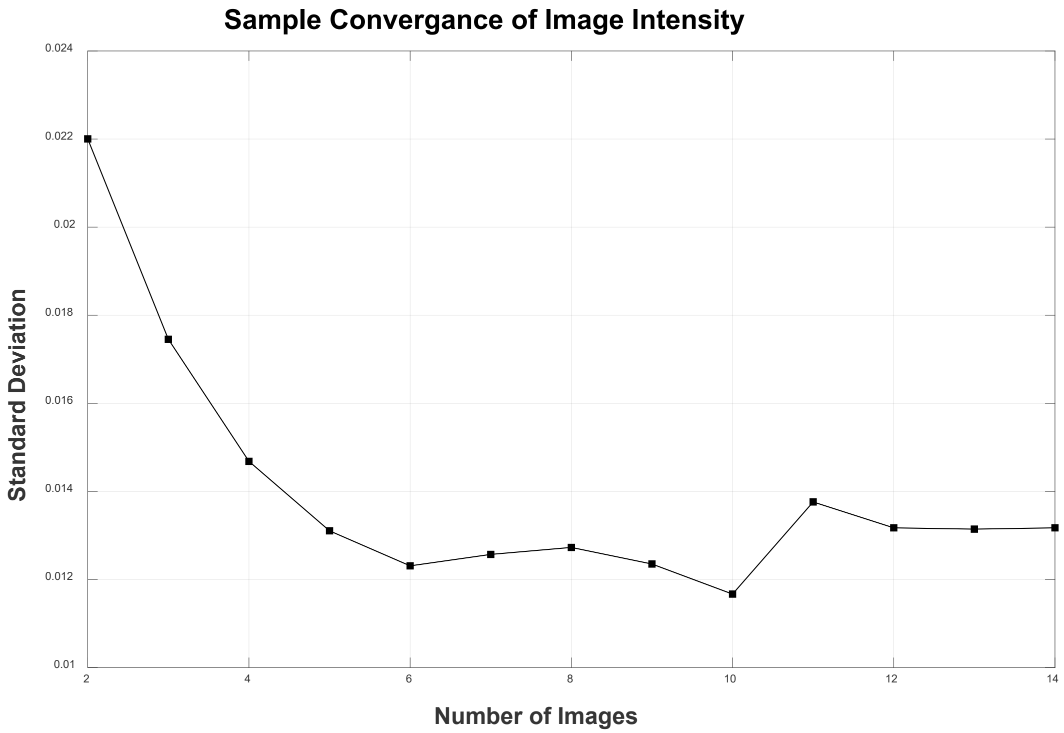

<u>Supplemental Figure S2.</u> Convergence of image intensity standard deviation.
To determine the number of images to acquire from each gel, 14 images from a single gel were obtained at random locations. The convergence of the standard deviation of average image intensity was determined by a simple difference algorithm. This procedure was repeated over 1,000,000 permutations of the order of images. The average convergence point was calculated to be 4.7 images. Thus, a minimum of six images were acquired from each gel to ensure sufficient sampling.

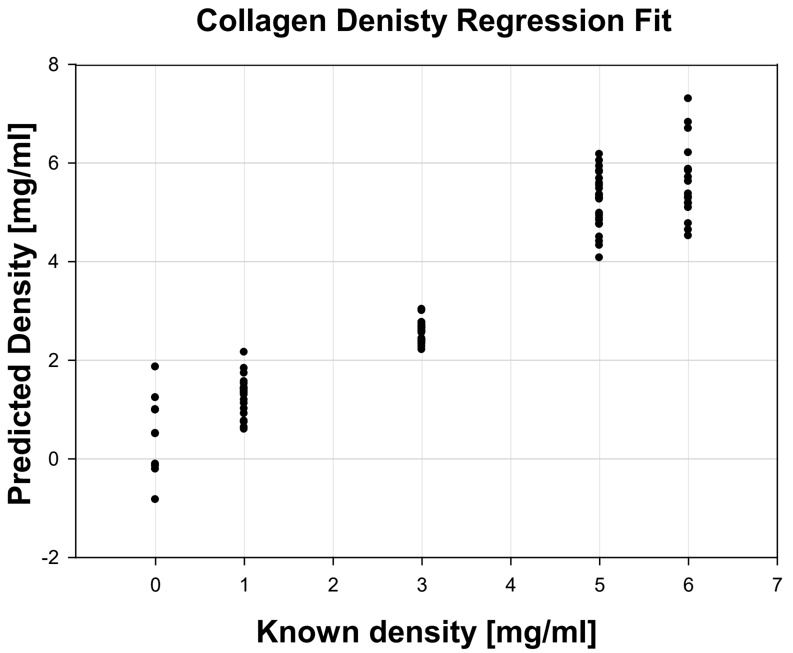

<u>Supplemental Figure S3.</u> Evaluation of collagen density regression.
The accuracy of multiple linear regression can be visualized by plotting the density predicted by the regression against known initial collagen concentration used experimentally. However, initial collagen concentration does not necessarily result in a homogenous density distribution, and this graph portrays the trend of capturing density with relative accuracy from 1-5 mg/ml.

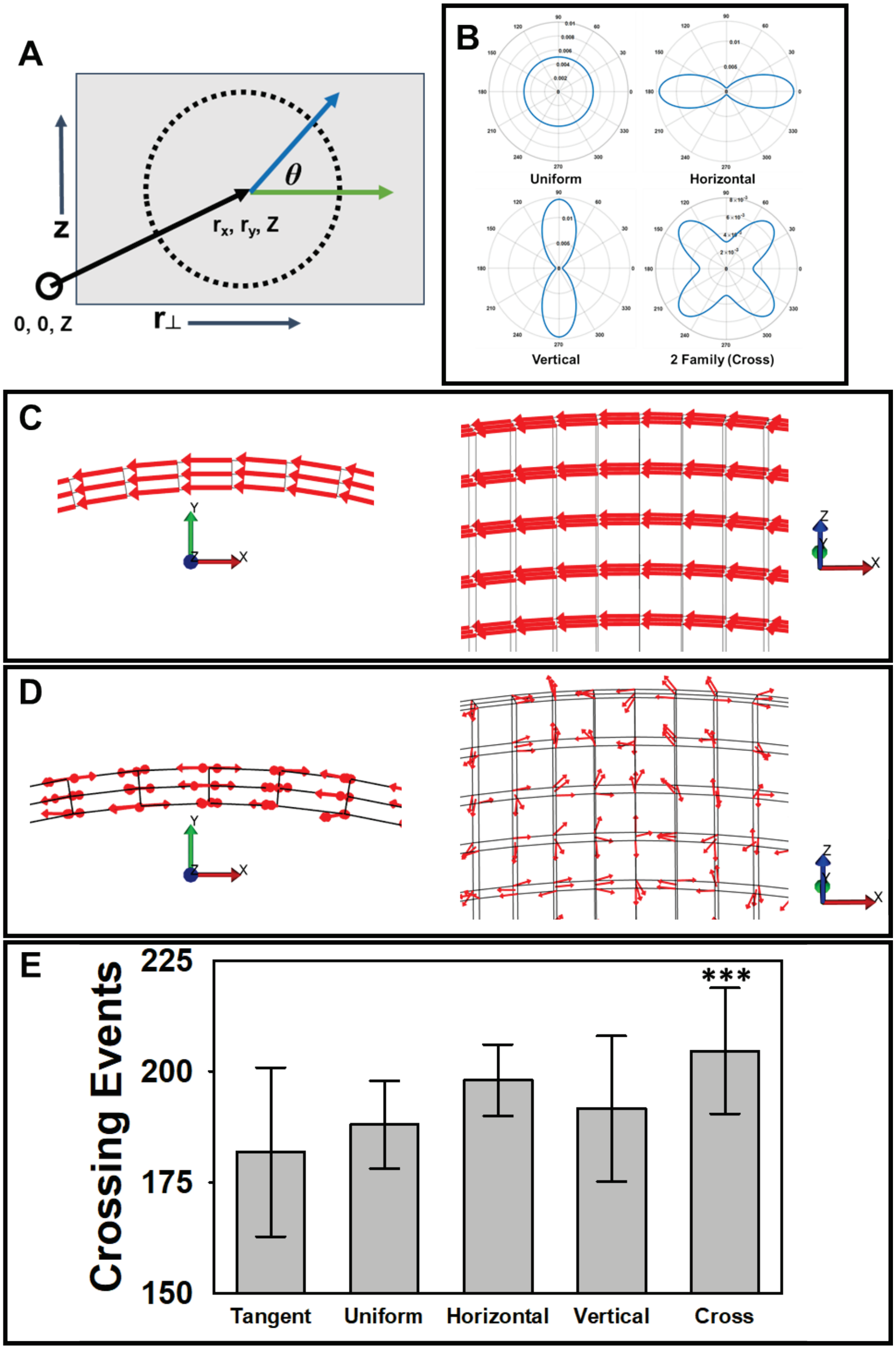

<u>Supplemental Figure S4.</u> Three-dimensional orientation of fibrils in the interface. Initially fibrils were assumed to have orientation in two dimensions, with two horizontal components but no vertical (Z) component. The distribution of ECM fibrils orientation at the interface was also considered, to determine if the frequency of vessel crossings relied on fibril

orientation. **A)** An interfacial element is shown in grey, and the element is tangent to the interface ($r\perp$, i.e. circumferential). A positional vector from the central axis to the center of the element is shown in black. The green vector represents the tangential vector that was studied in the 2D anisotropy mesh study; this vector lies tangent to the interface and has no vertical component. Tangential vectors were rotated by an angle θ (blue vector) about the axis of the positional vector so that they remain tangent to the interface, but contained a vertical component to introduce 3D orientation. **B)** Each element's fibril direction was rotated by an angle sampled from a von Mises probability distribution function. These polar plots are centered on the face of elements and lie in the $r\perp$-Z plane. Top left: A uniform distribution in which all angles of rotation are equally likely to be sampled. Top right: A distribution that favors alignment in the horizontal direction. Bottom left: A distribution that favors alignment in the vertical direction. Bottom right: A distribution of two fiber families that favor alignment at $\pm 45^{o}$. **C)** Vector plot of prescribed fibril alignment for the case of fibrils tangential to the interface with no vertical component. Left: Top-down view (XY plane). All fibrils lie tangential to the interface. Right: Side view of interface mesh and fibril orientation. All fibrils lie within the XY plane along the direction of their elements. **D)** Vector plots for prescribed fibril alignment biased in the vertical direction (**B**, bottom left). Left: When viewed from above all the fibril directions are still tangential to the interface. Right: A side angle view highlights the new 3D orientation that includes a vertical component. **E)** Crossing results: The mean and standard deviation for each model tested are displayed. A One-way ANOVA with Holm-Sidak all multiple comparison procedure identified significant differences only between tangential alignment (no vertical component) and the cross (2 family) distributions ($p = 0.008$, for ANOVA; $p < 0.001$, for the significant APMCP). The total crossing level remains high regardless of vertical fibril alignment.

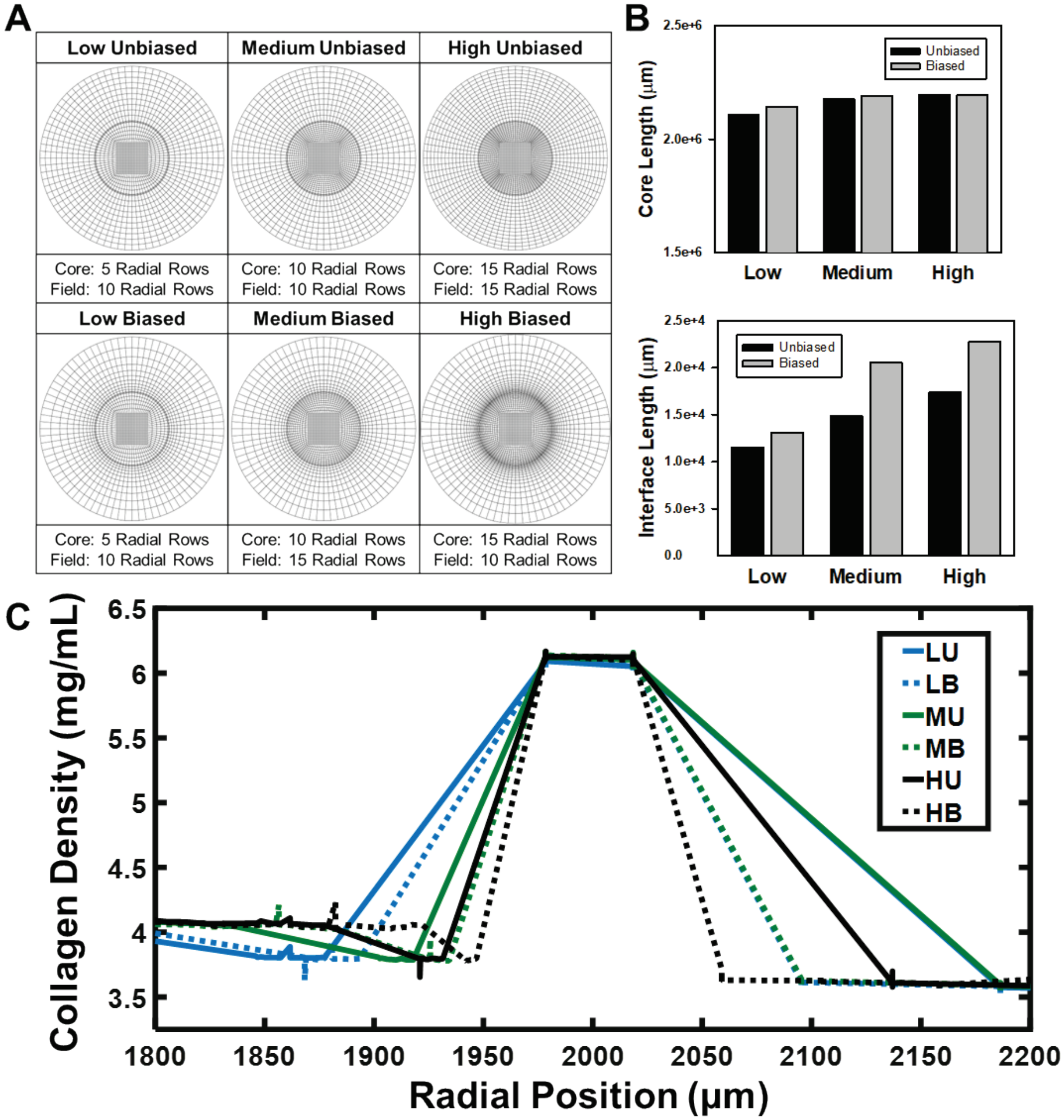

__Supplemental Figure S5.__ **Density gradient computational mesh study.**
A mesh study was performed to determine the radial meshing needed to provide an accurate spatial description of the local apparent collagen density in AngioFE. **A)** Meshes of low, medium, or high radial refinement and biasing were investigated. **B)** Comparison of vessel network length in the core to assess effect of meshing on growth. Core growth did not vary with mesh density and biasing. Interface growth increased with mesh refinement and biasing. The low refined meshes both predicted few (<10) crossings. None of the medium or highly refined meshes predicted growth in the fields. **C)** Average final collagen density as a function of the initial radial position (interface initially between 1960 – 2040 µm). The high biased mesh best represented the steep change in collagen density between the core and the interface whereas the

other schemes extend the range of the density gradient. The differences in predicted growth (B) are explained by the effective interface region, where a high biased mesh restricts interpolation effects to ~ 10 μm on each side of the interface. The high biased mesh was chosen for models used in the main body.

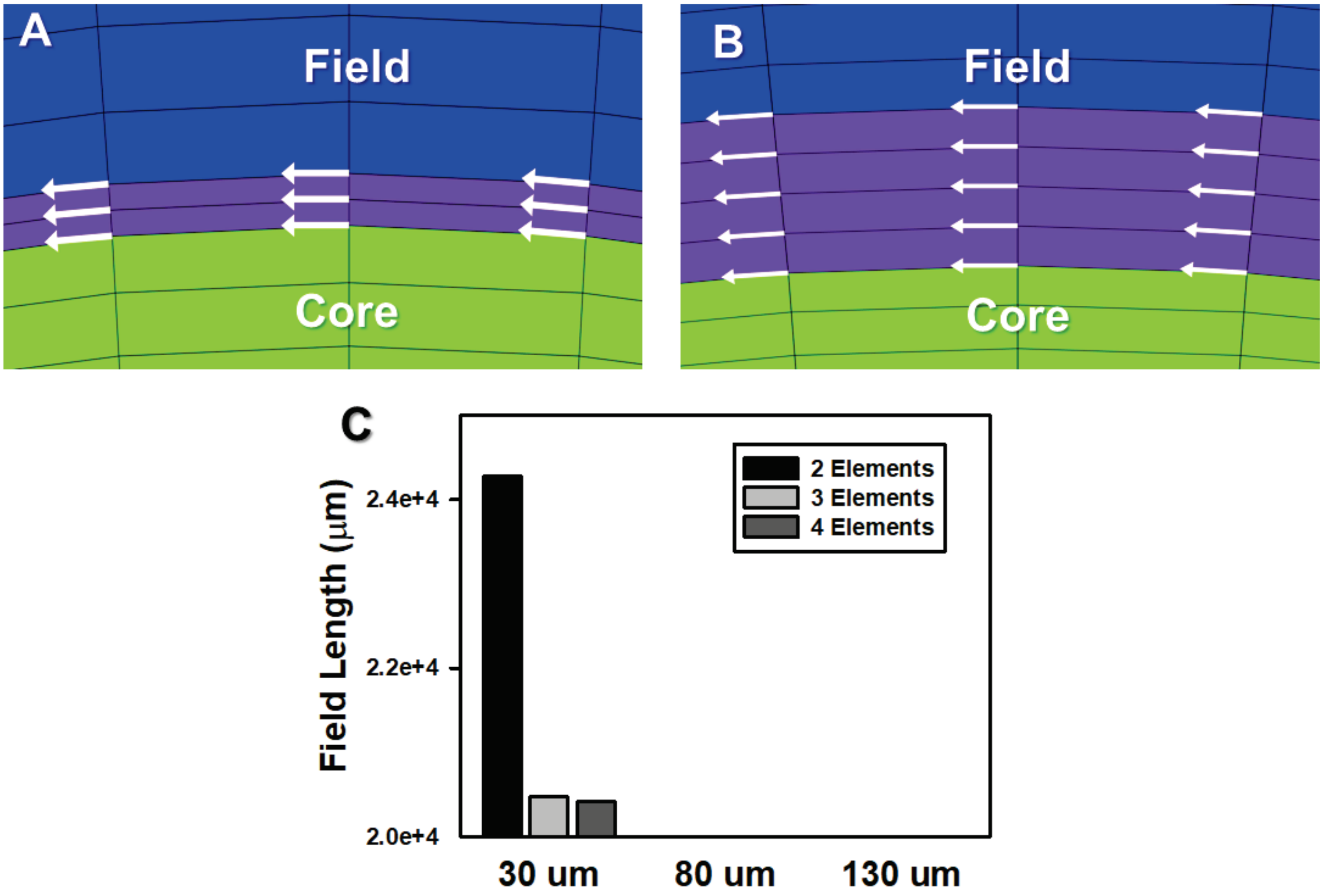

<u>Supplemental Figure S6.</u> Interface anisotropy computational mesh study.
A) Geometry and mesh for the case of a 30 µm thick interface with 2 radial elements. The core is green, interface purple, and field blue. The local fibril directions are projected to the nodes and represented as arrow glyphs. **B**) Geometry and mesh for the case of a 130 µm thick interface with 4 radial elements. **C**) Total vessel network length in the field for each simulated mesh. Bars are grouped by mesh thickness. The 30 µm thick interface failed to prevent crossing in the case of complete interfacial alignment and reliance of growth direction on fibril direction. Other thicknesses completely abrogated vessel crossing.

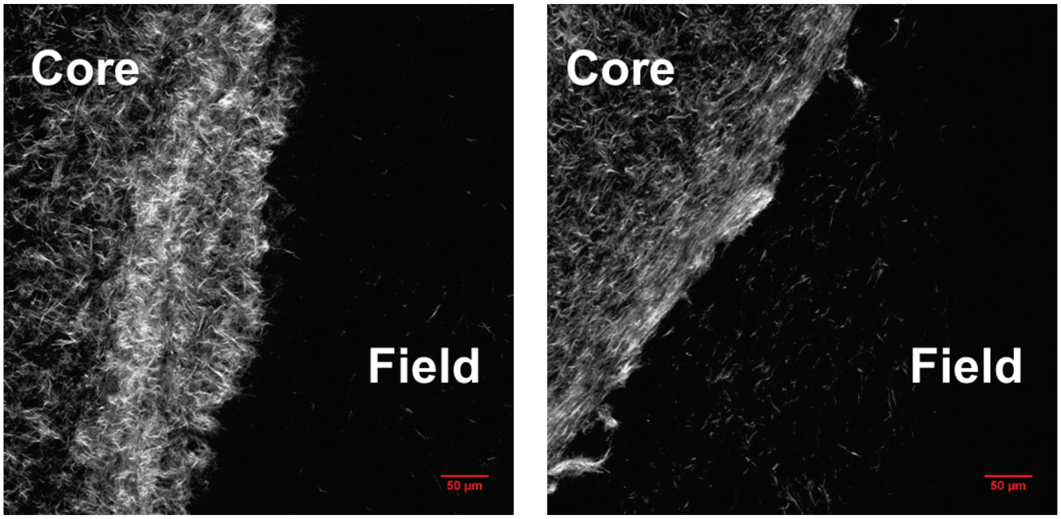

<u>**Supplemental Figure S7.**</u>
SHG images of fibril orientation at the interface. Orientation was inconsistent and varied between isotropic (left) and slight alignment (right). Visible changes in intensity between the core and field highlight the differences in density.

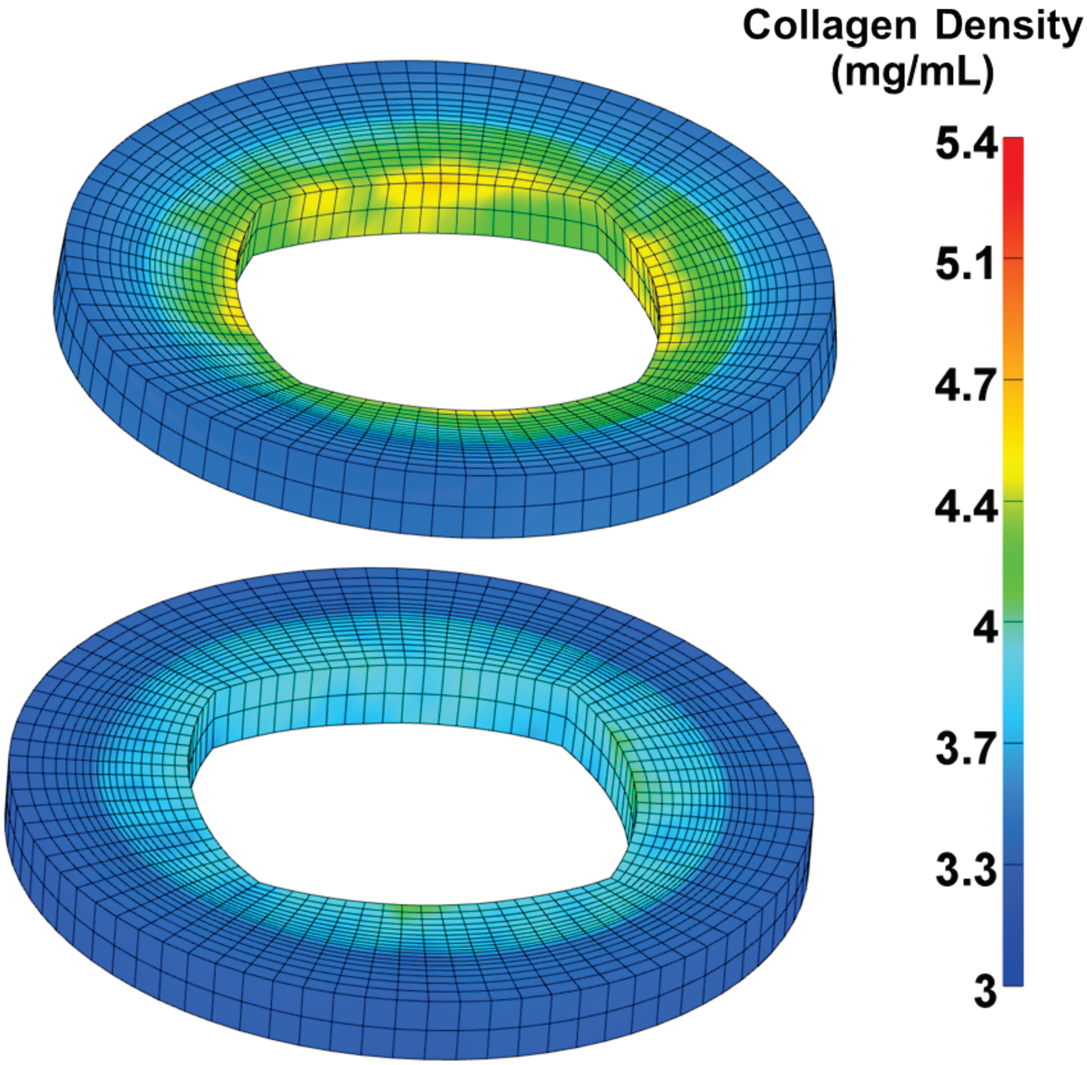

<u>Supplemental Figure S8.</u> Density sampling regions.
When imaging collagen gels using SHG to determine the local collagen density, we only imaged within a 400 μm diameter around the interface restricted to depths between 100-300 μm from the top and bottom face of the gels. Density measures from simulations came from elements in similar z positions as shown above.

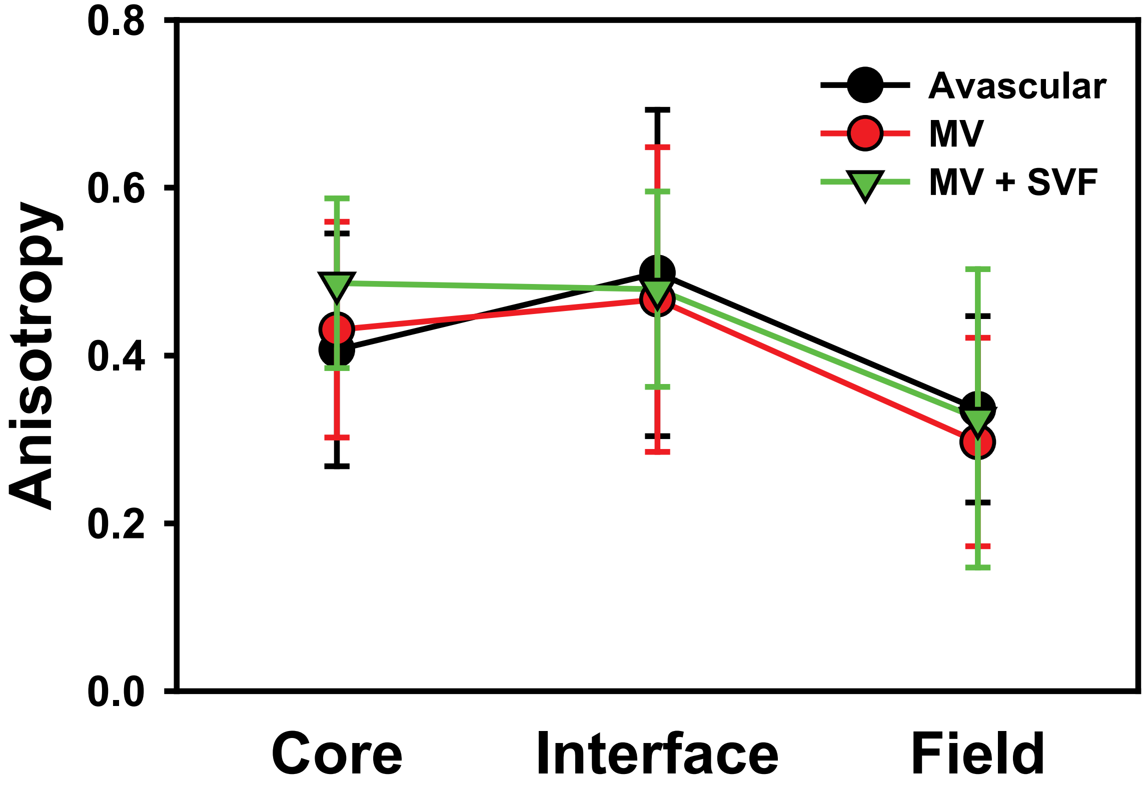

Supplemental Figure S9. Variations in anisotropy by gel region.
Study on interaction between gel region and cell fraction. A value of 0.0 indicates isotropy while a value of 1.0 indicates uniaxial alignment. The field was less anisotropic than the core and interface regardless of cell population.

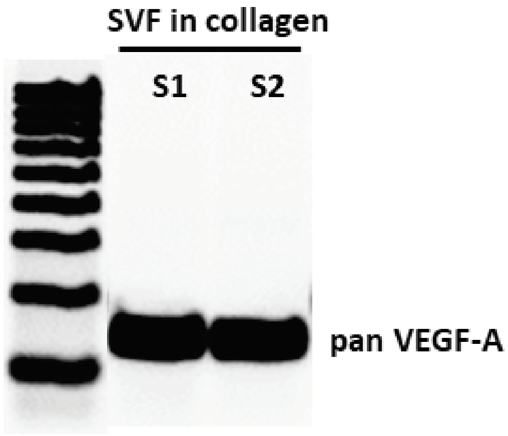

<u>Supplemental Figure S10.</u> VEGF expression in cultured SVF.
PCR of pan-VEGF transcripts from two samples of SVF cultured in collagen gels, demonstrating SVF production of VEGF.

3 Supplement References

Edgar LT, Hoying JB, Utzinger U, Underwood CJ, Krishnan L, Baggett BK, Maas SA, Guilkey JE and Weiss JA. Mechanical interaction of angiogenic microvessels with the extracellular matrix. *J Biomech Eng*. 2014;136:021001.

Edgar LT, Hoying JB and Weiss JA. In Silico Investigation of Angiogenesis with Growth and Stress Generation Coupled to Local Extracellular Matrix Density. *Annals of biomedical engineering*. 2015;43:1531-42.

Maas SA, Ellis BJ, Ateshian GA and Weiss JA. FEBio: finite elements for biomechanics. *J Biomech Eng*. 2012;134:011005
